# Astodrimer Sodium Nasal Spray versus Placebo in Non-Hospitalised Patients with COVID-19: A Randomised, Double-Blinded, Placebo-Controlled Trial

**DOI:** 10.3390/pharmaceutics16091173

**Published:** 2024-09-06

**Authors:** Stephen Winchester, Alex Castellarnau, Kashif Jabbar, Meera Nadir, Kapila Ranasinghe, Xavier Masramon, George R. Kinghorn, Isaac John, Jeremy R. A. Paull

**Affiliations:** 1 Frimley Health National Health Service Foundation Trust, Camberley GU16 7UJ, UK; s.winchester@nhs.net; 2 Starpharma Pty Ltd., Abbotsford, VIC 3067, Australia; alex.castellarnau@starpharma.com; 3 Ashford and St. Peter’s Hospitals National Health Service Foundation Trust, Chertsey KT16 0PZ, UK; kashif.jabbar@nhs.net (K.J.); meera.nadir@nhs.net (M.N.); kapila.ranasinghe@nhs.net (K.R.); isaac.john@nhs.net (I.J.); 4 Servicio de Asesoría a la Investigación y Logística (SAIL), 08027 Barcelona, Spain; xavier.masramon@sail-biometria.com; 5 Royal Hallamshire Hospital, University of Sheffield, Western Bank, Sheffield S10 2TN, UK; g.r.kinghorn@sheffield.ac.uk; 6 Royal Holloway, University of London, Egham TW20 0EX, UK

**Keywords:** astodrimer, COVID-19, dendrimer, antiviral, SARS-CoV-2, SPL7013, intranasal spray

## Abstract

Background/Objectives: Dendrimer-based astodrimer sodium nasal spray was assessed for its ability to reduce SARS-CoV-2 load in outpatients with COVID-19, which remains a severe illness for vulnerable groups. Methods: This was a randomised, double-blind, placebo-controlled clinical investigation evaluating the efficacy of astodrimer nasal spray in reducing SARS-CoV-2 viral burden in the nasopharynx of outpatients with COVID-19. Non-hospitalised adults with SARS-CoV-2 infection were randomised 1:1 to astodrimer or placebo four times daily from Day 1 to Day 7. Nasopharyngeal swabs for SARS-CoV-2 load determination were self-obtained daily from Day 1 to Day 8. The primary endpoint was an area under the curve of SARS-CoV-2 RNA copies/mL through Day 8 (vAUC_d1–8_). The primary analysis population was the modified intent-to-treat population (mITT: all randomised participants exposed to the study treatment who had at least one post-baseline viral load determination). Safety analyses included all randomised participants exposed to the study treatment. Study registration: ISRCTN70449927; Results: 231 participants were recruited between 9 January and 20 September 2023. The safety population comprised 109 and 113 participants randomised to astodrimer and placebo, respectively, with 96 and 101 participants in the mITT. Astodrimer sodium nasal spray reduced the SARS-CoV-2 burden (vAUC_d1–8_) vs. placebo in non-hospitalised COVID-19 patients aged 16 years and over (−1.2 log_10_ copies/mL × Day). The reduction in SARS-CoV-2 load was statistically significant in those aged 45 years and older (−3.7, *p* = 0.017) and the effect increased in older age groups, including in those aged 65 years and older (−7.3, *p* = 0.005). Astodrimer sodium nasal spray increased the rate of viral clearance and helped alleviate some COVID-19 symptoms, especially loss of sense of smell. Overall, 31 participants (14%) had ≥1 adverse event (AE). Four AEs were deemed possibly related to treatment. Most AEs were of mild severity and occurred at similar rates in both treatment arms. Conclusions: Astodrimer nasal spray reduces viral burden and accelerates viral clearance, especially in older populations, and is well tolerated.

## 1. Introduction

The magnitude of the infecting dose of virus an individual is exposed to can influence the transmissibility and severity of respiratory viral infections [1]. Studies in animal models have shown that increasing the viral challenge results in increased infection frequency and illness severity [2,3]. Human studies have demonstrated a positive correlation between infecting dose and severity of influenza [4]. A higher SARS-CoV-2 load is associated with increased disease severity and mortality [5,6,7] as well as transmissibility [8,9].

COVID-19 remains a severe condition for vulnerable groups including the elderly, unvaccinated or immunocompromised individuals, and those with respiratory comorbidities [10]. Strategies to reduce viral exposure are needed to complement preventive and therapeutic options and help lessen the incidence and severity of COVID-19 as well as other potential pandemic respiratory infections.

The nose is a key entry point for airborne viruses [11,12,13], with the viral load at its highest in the nasopharynx during early SARS-CoV-2 infection. A temporary nasal barrier to reduce viral load early in infection may limit or prevent illness.

**Figure 1:**
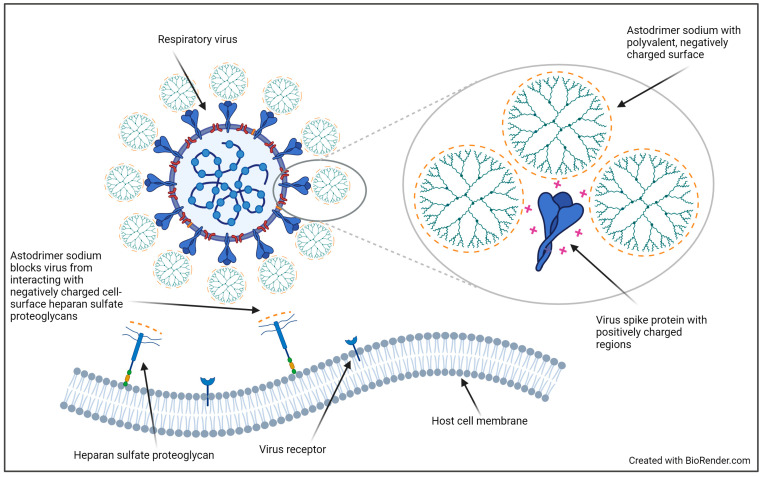
Representation of astodrimer sodium dendrimer structure and mechanism of antiviral action.

Astodrimer sodium is a large, negatively charged polylysine dendrimer with antiviral activity against a broad spectrum of viruses, including SARS-CoV-2 variants of concern, as demonstrated in vitro [14,15] and in vivo [16]. Astodrimer sodium has a polyvalent structure that enables multiple engagement of the positively charged regions of viral surface or spike proteins that interact with negatively charged host–cell surface heparan sulphate proteoglycans (HSPGs) to initiate the infection process. The polyvalent electrostatic interaction of astodrimer sodium with the virus attachment proteins is effectively irreversible, leading to inactivation of the virus and elimination of its ability to interact with the host–cell surface HSPGs (Figure 1).

Astodrimer sodium has been formulated as a nasal spray to trap and thereby inactivate viruses in the nasal cavity, reducing exposure to infectious virus and enhancing viral clearance. The safety and tolerability of astodrimer sodium nasal spray and lack of systemic absorption were previously demonstrated in healthy volunteers who used the product four times daily for 14 days [17].

The aims of this randomised clinical investigation were to evaluate the efficacy of astodrimer sodium nasal spray compared to placebo in reducing SARS-CoV-2 viral burden in the nasopharynx of patients with mild to moderate COVID-19 and to confirm the safety and tolerability of the nasal spray in patients with an active respiratory infection. Astodrimer sodium nasal spray reduced SARS-CoV-2 burden and increased viral clearance and was well tolerated.

## 2. Materials and Methods

### 2.1. Study Design

This was a single-centre, double-blinded, randomised, placebo-controlled, confirmatory clinical investigation of the efficacy and safety of astodrimer sodium nasal spray in the treatment of outpatients with COVID-19. The study was conducted at St Peter’s Hospital (Chertsey, UK) in accordance with all applicable guidelines and regulations, including the ICH and ISO Guidelines for Good Clinical Practice, and the Declaration of Helsinki. Ethics approval was obtained from the Leicester Central Research Ethics Committee on 23 November 2022 and the National Health Service (NHS) Health Research Authority on 28 November 2022 and the study was registered on isrctn.com (ISRCTN70449927).

### 2.2. Participants

Eligible participants were non-pregnant, non-breastfeeding individuals aged 16 years and older with a confirmed active COVID-19 infection by a positive PCR test within two days of enrolment. Those with a pending PCR test result could be randomised with a positive rapid antigen test (RAT). Participants whose baseline PCR test results were negative after enrolment were withdrawn. Participants needed a WHO COVID-19 clinical progression scale (CPS) score of one to three, indicating no need for hospitalisation [18]. Known allergies to astodrimer sodium or ingredients in the nasal spray and use of inhalation or nasal route medications (except for asthma or COPD treatments) were exclusion criteria.

### 2.3. Randomisation and Masking

Two hundred patients were planned to be randomised in a 1:1 ratio to receive astodrimer sodium nasal spray or an identically preserved saline placebo nasal spray. The randomisation list was computer generated based on a permutation block procedure and linked the treatment group with the unique randomisation numbers. Blinding was achieved using identical delivery devices, each consisting of an opaque bottle and a nasal pump. Treatment allocation was accomplished by identifying each delivery device with both a unique product number and the unique participant randomisation number. The site pharmacist dispensed study treatments per the randomisation list.

### 2.4. Procedures

After consent and, if applicable, collecting a nasopharyngeal swab for a SARS-CoV-2 RAT, participants were randomised and assigned a study treatment. Participants self-applied one pump of nasal spray (delivering a nominal volume of 100 µL) per nostril four times daily at evenly spaced intervals from Day 1 until either the end of Day 7 or until disease progression to a WHO CPS score of ≥4 (i.e., hospitalisation) [18], whichever occurred first. Those completing the 7-day treatment attended a final visit on Day 8.

Nasopharyngeal swabs were self-collected daily from Day 1 (baseline) to Day 8. The baseline sample was collected before the first application; subsequent samples were taken in the morning before the day’s first nasal spray use. The final sample was obtained during the Day 8 visit.

Participants were given access to an electronic diary (Greenlight Guru Clinical, Indianapolis, IN, USA), which they used daily to record details of nasopharyngeal swab sampling, nasal spray application, adverse events (AEs), concomitant medications, and to complete the inFLUenza Patient-Reported Outcome Plus (FLU-PRO^©^ Plus) symptoms questionnaire [19,20].

**Figure 2:**
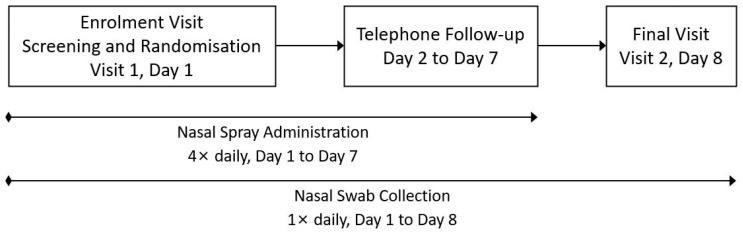
Schematic of study design.

Figure 2 presents a schematic of the study design.

### 2.5. Outcomes

#### 2.5.1. Primary Performance Outcome

The primary objective was to demonstrate the treatment effect of astodrimer sodium nasal spray in reducing the SARS-CoV-2 viral burden in the nasopharynx, measured by the area under the curve (AUC) from Day 1 to Day 8 of the logarithm of nasal swab SARS-CoV-2 RNA copies per mL (vAUC_d1–8_).

#### 2.5.2. Secondary Performance Outcomes

Secondary objectives included evaluating the efficacy of the nasal spray in reducing SARS-CoV-2 in the nasopharynx, preventing disease progression, shortening the time to negative conversion, and improving symptoms. Endpoints included time to overall recovery from symptoms, incidence of hospitalisation, time to negative RT-qPCR, proportion with negative RT-qPCR by Day 8, peak post-baseline viral load, and time to peak viral load.

Exploratory outcomes included viral clearance rate, viral load at each determination, and mean change in viral load relative to baseline. Exploratory endpoints related to symptoms were evaluated daily, including FLU-PRO total and domain scores, the proportion of symptomatic and recovered participants based on these scores, and the proportion with loss and recovery of smell and/or taste. A further set of endpoints explored the time to recovery from symptoms, the time taken to regain a lost sense of smell or taste, and the time until participants who reported an inability to perform their usual activities could return to those activities.

#### 2.5.3. Safety Outcomes

Safety and tolerability were assessed by documenting AEs and serious AEs (SAEs). AEs and medical history conditions were coded using MedDRA version 26.1. Concomitant medications were coded using WHODRUG 2023.

### 2.6. Statistical Analysis

Statistical analyses were detailed in a clinical investigation plan and a statistical analysis plan. Analyses were conducted using SAS^®^ version 9.4 in a secure and validated environment. 

#### 2.6.1. Analysis Populations

Primary, secondary and exploratory analyses were conducted on the modified intent-to-treat (mITT) population, which included randomised participants who received at least one dose of the study product and had at least one post-baseline viral load determination. Participants with a negative baseline RT-qPCR result were excluded from the mITT. Based on previously reported evidence that age is directly related to SARS-CoV-2 viral load [21], the primary analysis and key secondary and exploratory analyses were also conducted in age-based subsets of the mITT population. These subgroups included participants aged 40 years and older (40 Y+), 45 Y+, 50 Y+, 55 Y+, 60 Y+ and 65 Y+. The safety population comprised randomised participants who received at least one nasal spray application.

#### 2.6.2. Primary Efficacy Analysis

The vAUC from Day 1 through Day 8 was calculated using viral load data expressed as log_10_ RNA copies per mL, applying the linear trapezoidal rule. This method required the imputation of missing data to ensure a complete set of daily viral load measurements was available. A mixed-effect model for repeated measures (MMRMs) was implemented to perform the necessary imputations.

The null hypothesis posited that the vAUC of nasal swab SARS-CoV-2 was the same for both the astodrimer sodium and the placebo groups. The alternative hypothesis stated that the mean vAUCs were different. The primary analysis employed an analysis of covariance (ANCOVA) model with two fixed-effect terms: the treatment group and the baseline viral load as a covariate. The least squares mean (LSM) difference between the astodrimer sodium (T) and placebo (P) groups was obtained, with *p*-values and 95% CI provided. If the 95% CI upper bound of the LSM difference was less than zero and the *p*-value was equal or less than 0.05, astodrimer sodium was deemed superior in reducing the SARS-CoV-2 burden.

Further details on how the viral load data were processed, the MMRMs, and the ANCOVA model are provided in the Supplementary Materials.

#### 2.6.3. Secondary and Exploratory Efficacy Analyses

Continuous and binary endpoints were summarised by treatment group and over time. Continuous endpoint differences were evaluated using Student’s *t*-test or the Mann–Whitney test, while intra-group changes were assessed using paired *t*-test or Wilcoxon signed-rank test. Binary endpoint differences were assessed using Fisher’s exact test and intra-group changes were assessed using McNemar’s test. FLU-PRO scores were compared using ANCOVA with baseline scores as a covariate. Time-to-event variables used the Kaplan–Meier method for survival plots and the log-rank test for group comparisons, with Cox proportional hazards model calculating hazard ratios (HRs). Statistical significance was set at α ≤ 0.05.

#### 2.6.4. Safety Analyses

Safety analyses were descriptive, summarising the number and percentage of participants who experienced AEs/SAEs by treatment group, system organ class, preferred term, severity and relationship to treatment.

#### 2.6.5. Sample Size Determination

Based on a previous study comparing nitric oxide nasal spray vs. placebo in COVID-19 patients [22], it was assumed that the mean difference in vAUC_d1–8_ between treatment arms and the combined standard deviation (SD) would be at least 4.5 and 8.65 log_10_ copies/mL × Day, respectively. Under this assumption, a sample size of 78 participants per group would yield 90% power in a two-sided *t*-test at α = 0.05. Consequently, the initial plan was to recruit a total of 160 patients (80 per group).

However, given potential differences in viral variants and study design, the initial sample size calculation was validated via a pre-planned blinded sample size recalculation conducted after 73 participants completed the investigation.

The recalculated sample size was estimated to provide up to 90% power to detect a 4.5 log_10_ copies/mL × Day difference at α = 0.05, with an increased combined SD of 9.80 log_10_ copies/mL × Day. An absence of correlation between the ANCOVA unadjusted vAUC_d1–8_ and the baseline viral load was also assumed. This recalculation resulted in a target sample size of 200 participants (100 per group). The use of simple blind pooled variance estimators ensured no impact on the primary efficacy type I error rate, with no penalties applied to *p*-values or CIs in the final analysis.

## 3. Results

### 3.1. Populations and Baseline Data

**Figure 3:**
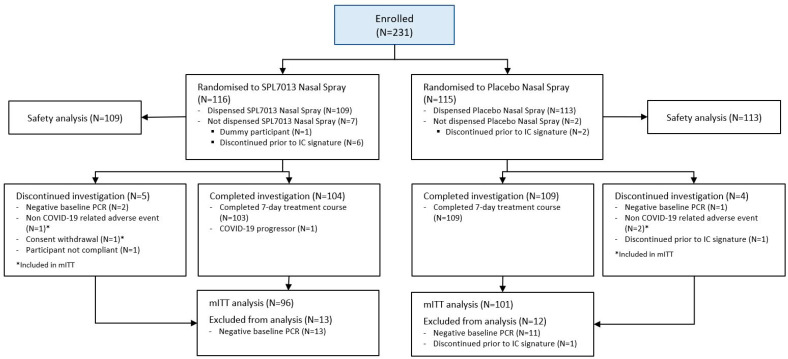
CONSORT flow diagram.

A total of 231 participants were enrolled in the study between 9 January and 20 September 2023 (astodrimer sodium nasal spray *n* = 116, placebo *n* = 115). The safety population comprised 222 participants (astodrimer *n* = 109, placebo *n* = 113). The mITT population comprised 197 participants (astodrimer *n* = 96, placebo *n* = 101). Further details of the analysis populations are presented in Figure 3.

The demographics and baseline characteristics of the mITT population are summarised in Table 1. Participants in both the astodrimer and placebo arms were of similar ages, with one-quarter aged 65 years or over. The astodrimer sodium group had a higher proportion of females (62.5%) compared to the placebo group (56.4%). The majority of participants were white. Both groups had similar heights, but the mean weight and body mass index (BMI) were slightly higher in the astodrimer group.

The enrolled population was highly vaccinated, with the majority of the mITT population (186 out of 197, 94.4%) having received at least one COVID-19 vaccination. On average, participants had received 3.5 COVID-19 vaccination doses each, with 131 reporting having received 3 or more doses (Table 2). The most frequent medical history conditions reported were vascular disorders (primarily hypertension), metabolic and nutritional disorders, neoplasms, respiratory disorders and gastrointestinal disorders. A history of diabetes was recorded in 12 participants (12.5%) receiving astodrimer and 6 (6%) receiving the placebo. A history of asthma or chronic obstructive pulmonary disease was recorded in 11 participants (11.5%) receiving astodrimer and 9 (8.9%) receiving placebo (Table S1). Concomitant medication use was reported by 158 participants (71.2%) in the mITT, with similar frequencies between groups. Direct-acting antivirals (nirmatrelvir/ritonavir or molnupiravir) were taken by four participants (3.7%) in the astodrimer sodium group and five participants (4.4%) in the placebo group.

### 3.2. Viral Load

**Figure 4:**
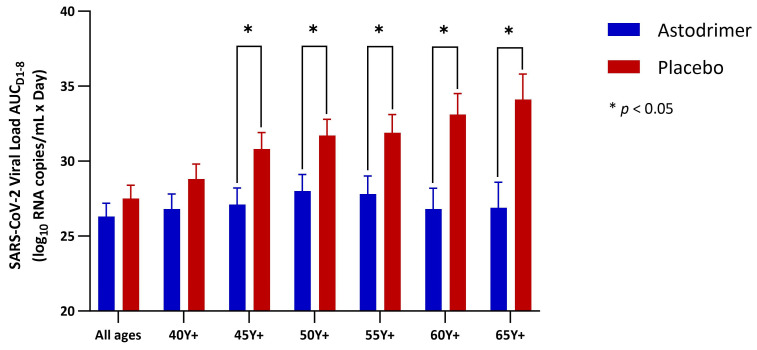
SARS-CoV-2 RNA load from Day 1 through Day 8 (vAUC_d1–8_) by treatment and age group. Compared using least squares means difference. * *p* < 0.05.

Results for the primary analysis are presented in Table 3 and Figure 4. The SARS-CoV-2 RNA burden (vAUC_d1–8_ for SARS-CoV-2 viral load) was lower in the astodrimer group than in the placebo group across the complete mITT (−1.2 log_10_ copies/mL × Day), although the difference did not reach statistical significance. However, the magnitude of the reduction in viral burden with astodrimer versus placebo increased with participant age and was statistically significant in the mITT age subgroups: 45 Y+ (−3.7 log_10_ copies/mL × Day, *p* = 0.017), 50 Y+ (−3.8 log_10_ copies/mL × Day, *p* = 0.018), 55 Y+ (−4.2 log_10_ copies/mL × Day, *p* = 0.024), 60 Y+ (−6.2 log_10_ copies/mL × Day, *p* = 0.003), and 65 Y+ (−7.3 log_10_ copies/mL × Day, *p* = 0.005).

**Figure 5:**
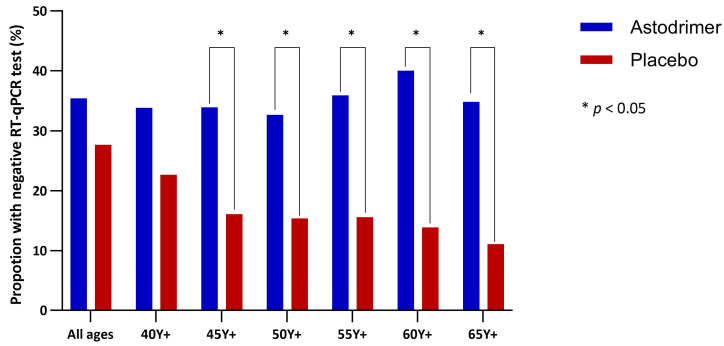
Proportion of participants with negative RT-qPCR test at or by Day 8, by treatment and age group. Compared using Fisher’s exact text. * *p* < 0.05.

**Figure 6:**
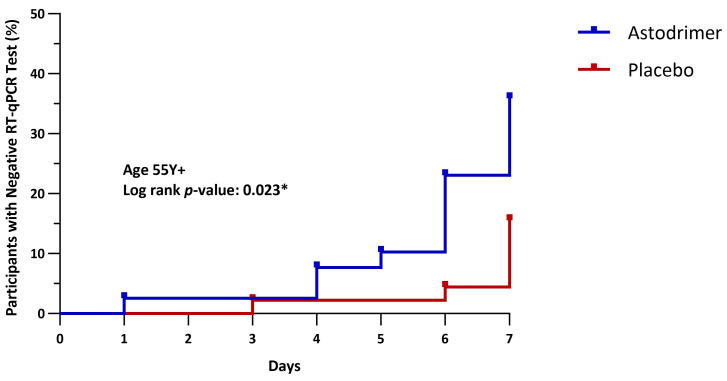
Time in days from baseline to negative RT-qPCR (55 Y+). Compared using log-rank test. * *p* < 0.05.

In the complete mITT population, a higher proportion of participants in the astodrimer group (35.4%, 34/96) attained a negative RT-qPCR test result at or by Day 8 compared to those assigned to the placebo group (27.7%, 28/101), although this difference did not reach statistical significance. However, beginning with the age subgroup 45 Y+, a significantly higher proportion of participants using astodrimer achieved a negative RT-qPCR compared to placebo (Figure 5). In these age subgroups, the negative RT-qPCR was attained in fewer days by those randomised to astodrimer, with statistically significant differences particularly pronounced in those aged 55 Y+ (Figure 6 and Figures S1–S7). Corresponding hazard ratios (HRs) were greater than two in the 45 Y+, 50 Y+, and 55 Y+ subgroups, and greater than three in the 60 Y+ and 65 Y+ subgroups (Table 4).

The peak SARS-CoV-2 RNA load post-baseline was marginally lower in the astodrimer group compared to the placebo group. Consistent with other results, the difference widened as participant age increased and reached statistical significance for participants aged 45 Y+, 55 Y+, 60 Y+, and 65 Y+ (Table S2, Figure S8). The time from Day 1 to peak viral load was slightly shorter in participants randomised to astodrimer, indicating an earlier start in the decline of viral load. The differences exhibited the same age-related pattern, ranging from 0.1 days in the complete mITT population to 0.4 days in the 65 Y+ subgroup (Table S3).

**Figure 7:**
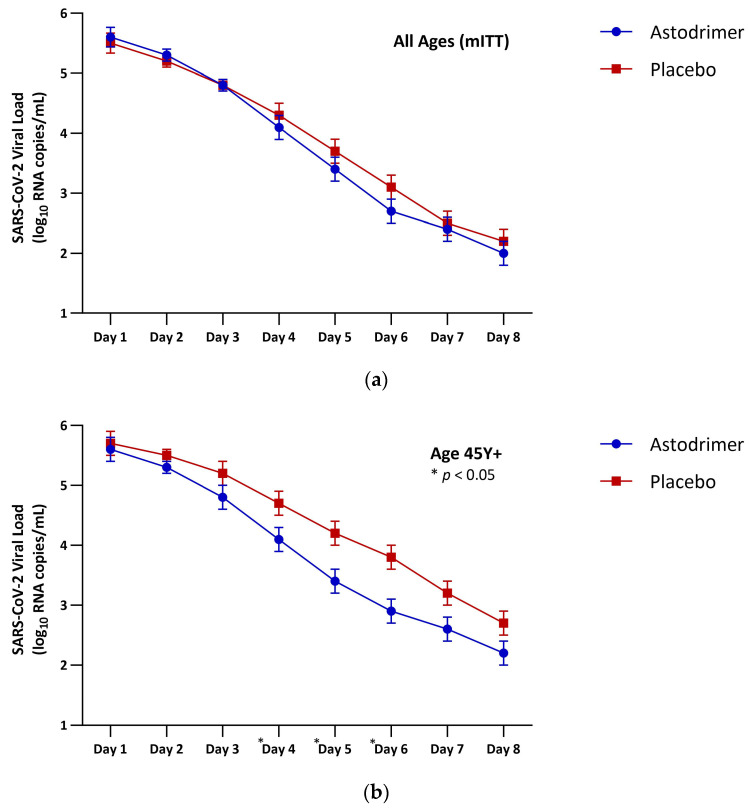

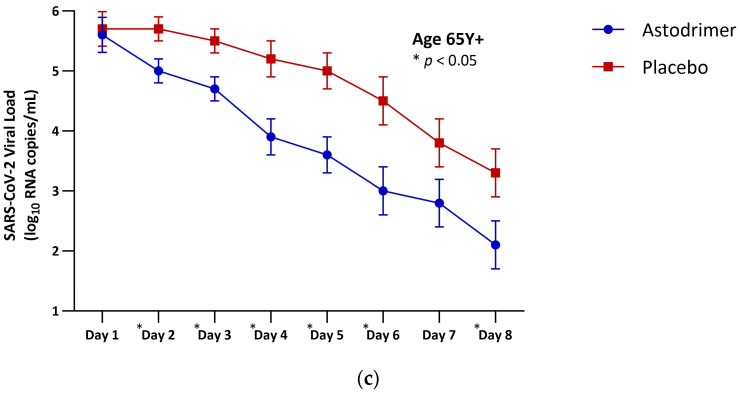
SARS-CoV-2 RNA load at each determination by treatment and age group: (**a**) all ages (mITT), (**b**) 45 Y+ and (**c**) 65 Y+. Compared using least squares means difference. * *p* < 0.05.

The SARS-CoV-2 clearance rate, determined by the slope of the decaying viral load curve from Day 2 to Day 6 and modelled using linear regression, was faster in the astodrimer group compared to the placebo group (−0.66 vs. −0.54, *p* = 0.035). Except for Day 1 and Day 2 in the complete mITT, where a slight baseline imbalance resulted in a marginally higher viral load in the astodrimer group, the viral load was lower in the astodrimer group at each daily determination (Figure 7a). These differences widened with age and became statistically significant for most study days beginning with the 45 Y+ subgroup (Figure 7b). Notably, participants randomised to astodrimer in the 65 Y+ subgroup demonstrated an 80% reduction in viral load relative to those using placebo on Day 2 after only two applications of the nasal spray (Figure 7c). Daily viral load values along with mean changes in viral load relative to the baseline determination are summarised in Supplementary Tables S4 and S5. The complete set of viral load curves is available as Supplementary Material.

### 3.3. Symptoms

FLU-PRO domain scores tended to be lower in the astodrimer arm, particularly among the older age groups. Participants aged 60 Y+ and 65 Y+ consistently showed lower symptom scores in the “eyes” domain (teary or watery, sore or painful eyes, and eyes sensitive to light), reaching statistical significance in several instances. Furthermore, a consistent trend favoured the astodrimer group over the placebo, with a lower proportion of symptomatic and a higher proportion of recovered participants noted at most assessment timepoints, with statistical significance reached for the “throat” (scratchy or itchy, sore or painful, swollen throat, and difficulty swallowing) and “eyes” symptom domains in some instances (Tables S6 and S7).

Of participants reporting symptoms on any one day after starting treatment, excluding gastrointestinal symptoms in participants aged 40 Y+, the proportion of participants recovered by Day 8 was consistently higher in the astodrimer group. In some instances, differences in recovery rates reached or approached statistical significance. Notably, within the “throat” domain, 80% of participants aged 55 Y+ in the astodrimer group recovered from symptoms, compared to 51.4% in the placebo group (*p* = 0.031); the differences in recovery rates for those aged 50 Y+ and 60 Y+ neared statistical significance (Table S8). Survival analysis of the time to recovery from COVID-19 symptoms indicated that among older participants, particularly in the “eyes” domain and the “throat” domain, the recovery time tended to be shorter for those randomised to astodrimer (Table S9).

There were no differences between groups in the time from Day 1 to overall recovery from symptoms. There was only one case of COVID-19 disease progression; this was a 71-year-old female participant randomised to astodrimer who was hospitalised on Day 6 of the investigation due to pneumonia.

No differences were observed in FLU-PRO total scores between the treatment arms at any post-baseline assessment. Similarly, there were no statistically significant differences in the proportion of symptomatic or recovered participants based on the total or domain symptom scores. 

### 3.4. Anosmia and Ageusia

**Figure 8:**
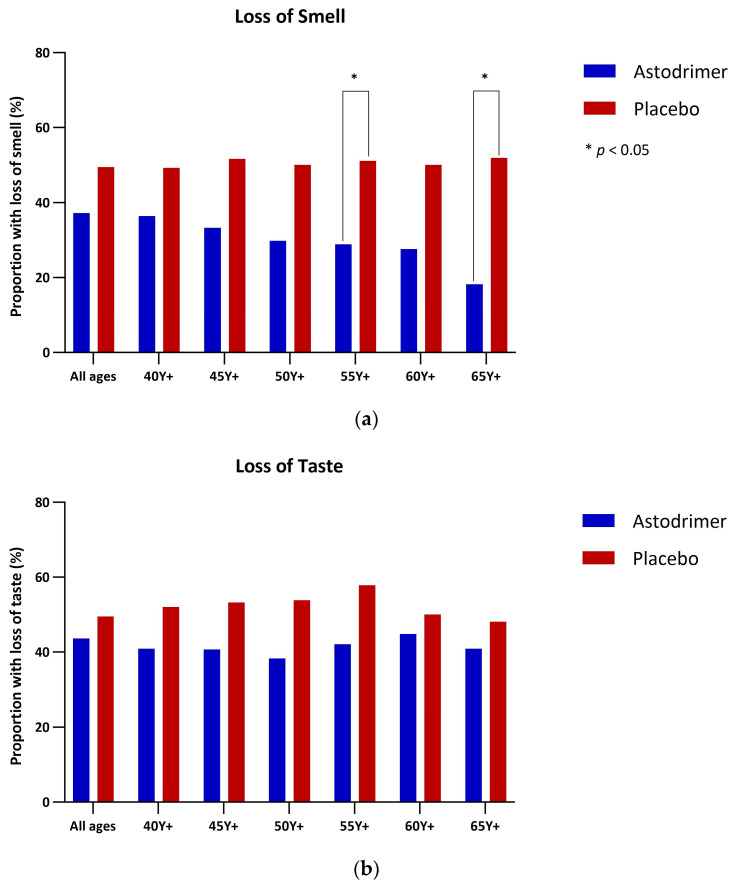

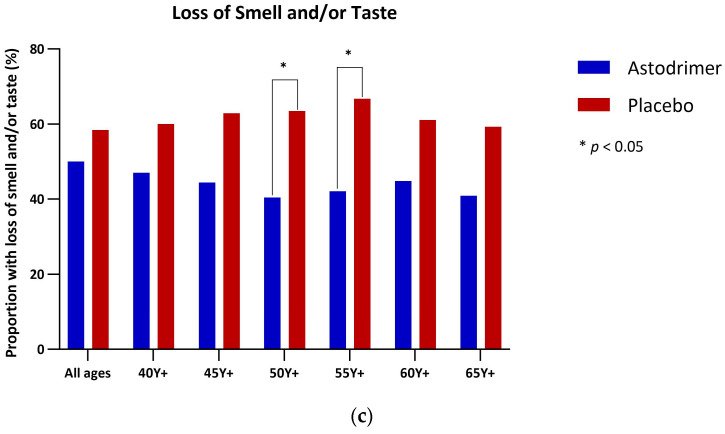
Proportion of participants with (**a**) loss of sense of smell (anosmia), (**b**) loss of sense of taste (ageusia) and (**c**) loss of sense of smell (anosmia) and/or taste (ageusia) on any given day after starting study treatment by age analysis group. Compared using Fisher’s exact text. * *p* < 0.05.

The proportion of participants with loss of sense of smell (anosmia) on any given day after starting the study treatment was consistently lower in the astodrimer sodium group (Table S10). This difference became more pronounced with advancing age, showing statistical significance in those aged 55 Y+ and 65 Y+ (Figure 8a). Similarly, the proportion of participants reporting loss of sense of taste (ageusia) was also consistently lower in those assigned to astodrimer, particularly among older population subsets (Figure 8b). The analysis of the proportion of participants reporting loss of sense of smell and/or loss of sense of taste followed the same pattern, with statistically significant results observed in the age subsets 50 Y+ and 55 Y+ (Figure 8c).

### 3.5. Return to Usual Activity

Participants in the astodrimer group, who had previously reported being unable to perform their usual activities due to COVID-19, returned to their activities one day earlier than those in the placebo group; however, this difference was not statistically significant.

### 3.6. Safety

Overall, 31 participants (14%) reported at least one adverse event (AE) during the study. The most common AEs were respiratory, thoracic and mediastinal disorders, occurring in six (5.5%) participants in the astodrimer group and three (2.7%) participants in the placebo group (Table S12). Four of the reported AEs were considered possibly related to the assigned nasal spray, including one case of epistaxis and two cases of nasal discomfort in the astodrimer group, and one case of administration site reaction in the placebo group. Generally, AEs were mild (Grade 1, 39 out of 45 events) and occurred at similar rates across both treatment arms. One participant from the astodrimer group and two from the placebo group experienced moderate AEs (abdominal pain, toothache, syncope), none of which were considered related to the nasal spray.

There were three severe and serious AEs, all deemed unrelated to the study product: one case of pneumonia in a participant given astodrimer and one case of urosepsis and one case of diabetic ketoacidosis, both experienced by the same placebo participant. These three events, along with one case of nasal discomfort, led to discontinuation of their participation in the study. Overall, astodrimer nasal spray was well tolerated, and adherence rates to the treatment regimen were high.

## 4. Discussion

In this clinical investigation, the use of astodrimer sodium nasal spray compared with placebo in patients with COVID-19 resulted in reduced SARS-CoV-2 RNA burden in the nasopharynx, accelerated viral clearance, a higher proportion of participants achieving a negative RT-qPCR test and in fewer days, lower peak viral load and an earlier start to the decline in viral load. The differences between groups became more pronounced with age, with statistically significant results observed in participants aged 45 years and older. These differences were particularly remarkable in older age groups, especially for those aged 60 and over and 65 and over, showing strongly statistically significant results (*p* < 0.01) despite the relatively smaller sample size in these subsets.

The study was not designed to detect differences in symptom-related measures, which were of exploratory nature. Nevertheless, consistent benefits associated with the use of astodrimer sodium nasal spray were observed. While differences from placebo were generally not statistically significant, the benefits for some outcomes, particularly in older age groups, did reach statistical significance. Symptom scores trended lower in the astodrimer group, with fewer symptomatic and more recovered participants noted at most assessment timepoints. Recovery from symptoms tended to be quicker for participants randomised to astodrimer. These observations were notable for throat- and eye-related symptoms. The proportion of participants experiencing a loss of sense of smell, taste, or both was consistently lower in those receiving astodrimer, from baseline through all subsequent assessments. Participants randomised to astodrimer generally reported taking less time to recover their lost sense of smell and taste, as well as a return to their usual activities in fewer days.

It is intuitive, and supported by many studies, that the magnitude of viral load can influence the transmissibility and severity of viral infections [1]. Viral load, along with viral clearance rate, is a clinically important determinant of the severity and prognosis of respiratory viral infections. Studies in cynomolgus macaques exposed to SARS-CoV-2 have demonstrated that variation in the infecting viral load dose determines how many animals are infected and how severe their illness is [3]. Human studies have shown a correlation between infectious viral load and the severity of illness due to the influenza virus [4]. Furthermore, several studies have indicated that SARS-CoV-2 viral load is associated with increased disease severity and mortality [5,6,7], as well as increased transmissibility of the infection [8,9]. Additionally, SARS-CoV-2 viral load detected in saliva has been shown to have potential prognostic value for the course of the disease [23]. Positive correlations between a patient’s age and viral load also suggest a rationale for the potential relationship between viral load and increased disease severity, with older age being established as a factor for worse COVID-19 outcomes [21]. The substantial body of evidence supporting the association and correlation of respiratory viral load with the severity of illness and the transmissibility of respiratory infections suggests significant potential and broad clinical relevance to the wider community of the results of the current study.

The study was conducted in a highly vaccinated and, consequently, well-protected population capable of overcoming the infection through their own immune response. Notably, there was only one case of COVID-19 disease progression requiring hospitalisation in the astodrimer arm and none in the placebo arm. Furthermore, at the time of enrolment, participants already had an active SARS-CoV-2 infection, with symptoms having commenced before starting the study treatment, suggesting that participants were already at or past the time of peak viral load, and the infection was already in its decline phase [24]. This scenario is inherent in studies of infectious diseases within the broader community, as it is not feasible to enrol participants and commence treatment at the exact moment of infection. In our study, the strong immune response, which was already underway when participants began treatment, significantly limited the observable effect associated with the use of astodrimer nasal spray. Nonetheless, we were able to demonstrate a more rapid viral clearance in the astodrimer group compared to placebo (*p* = 0.035) and better outcomes across all viral load measures, albeit those outcomes were not statistically significant for the complete analysis set.

Differences between groups became more pronounced with age, showing statistically significant benefits (*p* < 0.05) in population subsets aged 45 years and older, with the best outcomes observed in those aged 60 and over and 65 and over. This observation aligns with previous research indicating a positive correlation between a patient’s age and viral load [21]. Importantly, vaccines depend on the host’s immune response, which can be diminished in the elderly, leading to reduced efficacy [25]. In contrast, treatments like astodrimer, which act directly and do not rely on eliciting an immune response from the host, can provide complementary clinical benefits in these cases. The observed age-based efficacy pattern is not unique to this investigation. A subgroup analysis from a large retrospective study evaluating the effectiveness of nirmatrelvir with ritonavir in reducing severe COVID-19 and mortality demonstrated that treatment with these antivirals resulted in a lower risk (HR of 0.52) only in patients aged 60 years and over [26].

SARS-CoV-2 is known to penetrate the olfactory mucosa, causing alterations in smell and taste perceptions as it migrates to the central nervous system (CNS) via the cribriform plate along the olfactory tract or through vagal or trigeminal pathways [27,28]. CNS viral invasion results in long-term neurological complications known as Long-COVID [29,30]. The notably lower proportion of participants experiencing loss of sense of smell and/or taste in the astodrimer group observed in this study is of particular importance. It suggests that the coating of the nasal mucosa resulting from the spray application might have prevented viral colonisation at the neural–mucosa interfaces, thereby reducing COVID-19-associated sensorial alterations and, potentially, CNS invasion. This hypothesis is supported by preclinical observations in K18-hACE2 mice challenged with SARS-CoV-2, where brain viral invasion was completely blocked in animals treated with astodrimer, whereas viral RNA was detected in the brains of those treated with phosphate-buffered saline (PBS) [16].

Another limitation of this study is the absence of a pre-stated hypothesis to evaluate symptom-related outcomes and their exploratory nature. Nevertheless, the consistency observed across different symptom measures underscores the robustness of the results.

## 5. Conclusions

Astodrimer sodium nasal spray reduced SARS-CoV-2 burden and increased the rate of viral clearance in non-hospitalised COVID-19 patients aged 16 and over. The reduction in SARS-CoV-2 load was statistically significant in those aged 45 years and older. By reducing viral load, astodrimer sodium helped alleviate some COVID-19 symptoms, especially loss of sense of smell. It was well tolerated, with primarily mild AEs reported and little evidence of risk. Given the association of viral load with illness severity and transmissibility of respiratory infections, our findings suggest that astodrimer sodium nasal spray, with its broad-spectrum activity, has a potential role in disrupting and reducing viral replication, preventing or reducing virus dissemination into immune-privileged sites (sensory neurons/cerebrum), and preventing onward community transmission, especially in high-risk or vulnerable populations such as the elderly or immunocompromised.

## Figures and Tables

**Table 1:** Participant demography and baseline characteristics (mITT population).

	Astodrimer (*n* = 96)	Placebo (*n* = 101)	Total (*n* = 197)
Age (Years)	50 (16, 86)	52 (19, 86)	50 (16, 86)
≥40 years (40 Y+)	68 (70.8%)	75 (74.3%)	143 (72.6%)
≥45 years (45 Y+)	56 (58.3%)	62 (61.4%)	118 (59.9%)
≥50 years (50 Y+)	49 (51.0%)	52 (51.5%)	101 (51.3%)
≥55 years (55 Y+)	39 (40.6%)	45 (44.6%)	84 (42.6%)
≥60 years (60 Y+)	30 (31.3%)	36 (35.6%)	66 (33.5%)
≥65 years (65 Y+)	23 (24.0%)	27 (26.7%)	50 (25.4%)
Gender			
Female	60 (62.5%)	57 (56.4%)	117 (59.4%)
Male	36 (37.5%)	44 (43.6%)	80 (40.6%)
Ethnicity			
White	81 (87.1%)	79 (78.2%)	160 (82.5%)
Asian (Indian, Pakistani or Bangladeshi)	8 (8.6%)	11 (10.9%)	19 (9.8%)
Asian (Chinese)	1 (1.1%)	3 (3.0%)	4 (2.1%)
Other	1 (1.1%)	6 (5.9%)	7 (6.9%)
Height (cm)	168.6 (9.1)	168.5 (10.7)	168.6 (9.9)
Weight (kg)	82.0 (19.4)	75.4 (16.5)	78.6 (18.2)
BMI (kg/m^2^)	28.8 (6.0)	26.4 (4.7)	27.6 (5.5)

Data are median (min, max), *n* (%), or mean (SD). BMI = body mass index. Age subgroups 40 Y+, 45 Y+, 50 Y+, 55 Y+, 60 Y+, and 65 Y+ represent participants aged greater than or equal to the indicated age cut-off (e.g., 40 Y+ includes all participants aged 40 years and older).

**Table 2:** Summary of COVID-19 vaccination history (mITT population).

	Astodrimer	Placebo	Total
COVID-19 vaccination status	(N = 96)	(N = 101)	(N = 197)
Participants not vaccinated	4 (4.2%)	4 (4.0%)	8 (4.1%)
Participants with at least 1 vaccination	90 (93.8%)	96 (95.0%)	186 (94.4%)
Missing	2	1	3
Vaccination history	(N = 68)	(N = 83)	(N = 151)
Number of vaccinations per participant	3.5 (1.1)	3.4 (0.8)	3.5 (1.0)
Participants with 1 vaccination	2 (2.9%)	0 (0.0%)	2 (1.3%)
Participants with 2 vaccinations	7 (10.3%)	11 (13.3%)	18 (11.9%)
Participants with 3 vaccinations	29 (42.6%)	32 (38.6%)	61 (40.4%)
Participants with 4 vaccinations	18 (26.5%)	32 (38.6%)	50 (33.1%)
Participants with 5 vaccinations	10 (14.7%)	8 (9.6%)	18 (11.9%)
Participants with 6 vaccinations	1 (1.5%)	0 (0.0%)	1 (0.7%)
Participants with 7 vaccinations	1 (1.5%)	0 (0.0%)	1 (0.7%)
Type of vaccine			
Pfizer/BioNTech	61 (89.7%)	78 (94.0%)	139 (92.1%)
Oxford/AstraZeneca	33 (48.5%)	41 (49.4%)	74 (49.0%)
Moderna	17 (25.0%)	33 (39.8%)	50 (33.1%)

Data are *n* (%). The number of ‘participants with at least 1 vaccination’ refers to those who reported being vaccinated, regardless of whether they provided details on their vaccination history. The sample size (N) under ‘vaccination history’ refers to the number of participants who provided detailed information on their vaccination history. The ‘number of vaccinations’ refers to the average number of vaccinations per participant. The ‘type of vaccine’ refers to the proportions of each vaccine type in the complete dataset; the total exceeds 100%, as participants could have received more than one type of vaccine.

**Table 3:** SARS-CoV-2 RNA load from Day 1 through Day 8 (vAUC_d1–8_).

	Astodrimer	Placebo	Astodrimer Versus Placebo
All ages (mITT)			
N	96	101	197
LSM (SE)	26.3 (0.9)	27.5 (0.9)	−1.2 (1.2)
95% CI	24.6, 28.1	25.8, 29.2	−3.6, 1.2
*p*-value			0.344
Age 40 Y+ (mITT)			
N	68	75	143
LSM (SE)	26.8 (1.0)	28.8 (1.0)	−2.0 (1.4)
95% CI	24.8, 28.9	26.9, 30.8	−4.8, 0.8
*p*-value			0.159
Age 45 Y+ (mITT)			
N	56	62	118
LSM (SE)	27.1 (1.1)	30.8 (1.1)	−3.7 (1.5)
95% CI	24.9, 29.3	28.7, 32.9	−6.8, −0.7
*p*-value			0.017 *
Age 50 Y+ (mITT)			
N	49	52	101
LSM (SE)	28.0 (1.1)	31.7 (1.1)	−3.8 (1.6)
95% CI	25.7, 30.2	29.6, 33.9	−6.9, −0.7
*p*-value			0.018 *
Age 55 Y+ (mITT)			
N	39	45	84
LSM (SE)	27.8 (1.2)	31.9 (1.2)	−4.2 (1.8)
95% CI	25.2, 30.4	29.5, 34.4	−7.7, −0.6
*p*-value			0.024 *
Age 60 Y+ (mITT)			
N	30	36	66
LSM (SE)	26.8 (1.4)	33.1 (1.4)	−6.2 (2.0)
95% CI	23.9, 29.8	30.4, 35.8	−10.3, −2.2
*p*-value			0.003 *
Age 65 Y+ (mITT)			
N	23	27	50
LSM (SE)	26.9 (1.7)	34.1 (1.7)	−7.3 (2.5)
95% CI	23.2, 30.5	30.8, 37.5	−12.2, −2.3
*p*-value			0.005 *

Values expressed as log_10_ SARS-CoV-2 RNA copies/mL × Day. LSM = least squares mean; SE = standard error; CI = confidence interval; * *p* < 0.05. The LSM results from the application of an ANCOVA model with two fixed effect terms: treatment group and the log_10_ of viral load at Day 1 (baseline) as a continuous covariate.

**Table 4:** Proportion of participants with negative RT-qPCR at or by Day 8 and hazard ratios.

	Negative RT-qPCR at or by Day 8		Fisher’s Exact Test	Hazard Ratio
	Astodrimer	Placebo		
All ages	34/96 (35.4%)	28/101 (27.7%)	*p* = 0.157	1.31 (0.79, 2.15), *p* = 0.297
Age 40 Y+	23/68 (33.8%)	17/75 (22.7%)	*p* = 0.097	1.54 (0.82, 2.87), *p* = 0.180
Age 45 Y+	19/56 (33.9%)	10/62 (16.1%)	*p* = 0.021 *	2.24 (1.04, 4.81), *p* = 0.040 *
Age 50 Y+	16/49 (32.7%)	8/52 (15.4%)	*p* = 0.035 *	2.31 (0.99, 5.40), *p* = 0.053
Age 55 Y+	14/39 (35.9%)	7/45 (15.6%)	*p* = 0.029 *	2.64 (1.06, 6.54), *p* = 0.036 *
Age 60 Y+	12/30 (40.0%)	5/36 (13.9%)	*p* = 0.016 *	3.27 (1.15, 9.29, *p* = 0.026 *
Age 65 Y+	8/23 (34.8%)	3/27 (11.1%)	*p* = 0.047 *	3.41 (0.90, 12.87), *p* = 0.070 *

Proportions of participants with negative RT-qPCR results at or by Day 8 were compared using Fisher’s exact test. Hazard ratios were derived from a survival analysis of time (in days) from baseline to negative RT-qPCR, based on a Cox proportional hazards model, and are expressed relative to astodrimer with 95% confidence intervals (CI). * *p* < 0.05.

## Data Availability

The original contributions presented in the study are included in the article/Supplementary Material; further inquiries can be directed to the corresponding author.

## Supplementary Materials

The following supporting information can be downloaded at https://www.mdpi.com/article/10.3390/pharmaceutics16091173/s1: additional details on viral load data processing and statistical methods, as well as supplementary tables (Tables S1–S12) and figures (Figures S1–S22).

## Abbreviations

AE: Adverse event; AUC: area under the curve; ANCOVA: analysis of covariance; BMI: body mass index; CI: confidence interval; CNS: central nervous system; COPD: chronic obstructive pulmonary disease; COVID-19: coronavirus disease 2019; FLU-PRO: inFLUenza patient-reported outcomes; HR: Hazard ratio; HSPG: Heparan sulphate proteoglycan; ICH: International council for harmonisation; ISO: international organization for standardization; LSM: least squares mean; MedDRA: medical dictionary for regulatory activities; MMRMs: mixed-effects model for repeated measures; mITT: modified intent-to-treat population; NHS: national health service; PBS: phosphate-buffered saline; PCR: polymerase chain reaction; RAT: rapid antigen test; RNA: ribonucleic acid; RT-qPCR: reverse transcription quantitative polymerase chain reaction; SAE: serious adverse event; SARS-CoV-2: severe acute respiratory syndrome coronavirus 2; SD: standard deviation; SE: standard error; vAUC: viral area under the curve; vAUC_d1–8_: viral area under the curve from day 1 to day 8; WHO CPS: World Health Organization—clinical progression scale.
