# Supplementary material for: Astodrimer Sodium Nasal Spray versus Placebo in Non-Hospitalised Patients with COVID-19: A Randomised, Double-Blinded, Placebo-Controlled Trial
Source: Pharmaceutics. 2024 Sep 6;16(9):1173. doi: 10.3390/pharmaceutics16091173 (PMC11435287; doi:10.3390/pharmaceutics16091173)

Table of contents

Viral load data processing 3

Mixed model for repeated measures (MMRM) 3

Adjustment for baseline imbalances (ANCOVA) 3

InFLUenza Patient-Reported Outcome Plus (FLU-PRO© Plus) symptoms questionnaire 3

Supplementary tables 4

Table S1: Medical history 4

Table S2: Peak nasal swab SARS-CoV-2 RNA load post-baseline 9

Table S3: Time in days from Day 1 to peak nasal swab SARS-CoV-2 RNA load post-baseline 10

Table S4: Nasal swab SARS-CoV-2 RNA load at each determination 11

Table S5: Mean change in nasal swab SARS-CoV-2 RNA load at each determination relative to baseline (Day 1) 12

Table S6: Proportion of symptomatic and recovered participants as per the throat symptoms score 14

Table S7: Proportion of symptomatic and recovered participants as per the eyes symptoms score 15

Table S8: Proportion of symptomatic participants who recovered from COVID-19 symptoms at or by Day 8 as per the total and domain-based symptoms scores 16

Table S9: In participants reporting COVID-19 symptoms as per the total or domain-based symptom scores, time in days from Day 1 to recovery from symptoms 18

Table S10: Proportion of participants reporting loss of sense of smell and/or taste on any one day after starting with the study treatment 20

Table S11: Proportion of participants who recovered their lost sense of smell and/or taste 21

Table S12: Summary of adverse events 22

Supplementary figures 24

Time to negative RT-qPCR test 24

Figure S1: Time in days from baseline to negative RT-qPCR (All ages) 24

Figure S2: Time in days from baseline to negative RT-qPCR (40Y+) 24

Figure S3: Time in days from baseline to negative RT-qPCR (45Y+) 24

Figure S4: Time in days from baseline to negative RT-qPCR (50Y+) 25

Figure S5: Time in days from baseline to negative RT-qPCR (55Y+) 25

Figure S6: Time in days from baseline to negative RT-qPCR (60Y+) 25

Figure S7: Time in days from baseline to negative RT-qPCR (65Y+) 26

Peak post-baseline nasal swab SARS-CoV-2 RNA load 26

Figure S8: Peak post-baseline nasal swab SARS-CoV-2 RNA load 26

SARS-CoV-2 RNA load at each determination 26

Figure S9: SARS-CoV-2 RNA load at each determination (All ages) 26

Figure S10: SARS-CoV-2 RNA load at each determination (40Y+) 27

Figure S11: SARS-CoV-2 RNA load at each determination (45Y+) 27

Figure S12: SARS-CoV-2 RNA load at each determination (50Y+) 27
Figure S13: SARS-CoV-2 RNA load at each determination (55Y+) 28
Figure S14: SARS-CoV-2 RNA load at each determination (60Y+) 28
Figure S15: SARS-CoV-2 RNA load at each determination (65Y+) 28
Mean change in SARS-CoV-2 RNA load at each determination relative to the baseline determination 29
Figure S16: Mean SARS-CoV-2 RNA load change at each determination relative to baseline (All ages) 29
Figure S17: Mean SARS-CoV-2 RNA load change at each determination relative to baseline (40Y+) 29
Figure S18: Mean SARS-CoV-2 RNA load change at each determination relative to baseline (45Y+) 29
Figure S19: Mean SARS-CoV-2 RNA load change at each determination relative to baseline (50Y+) 30
Figure S20: Mean SARS-CoV-2 RNA load change at each determination relative to baseline (55Y+) 30
Figure S21: Mean SARS-CoV-2 RNA load change at each determination relative to baseline (60Y+) 30
Figure S22: Mean SARS-CoV-2 RNA load change at each determination relative to baseline (65Y+) 31

Viral load data processing

Prior to the statistical analyses, nasal swab SARS-CoV-2 viral load data, expressed as RNA copies per mL, underwent three processing steps. Results reported as negative were imputed to zero RNA copies per mL. Subsequently, each viral load value was incremented by one unit, so values previously imputed as zero copies per mL were reassigned to one copy per mL. Finally, the viral load data were subjected to a log_{10} transformation. The logarithmically transformed viral load data, expressed in terms of log_{10} RNA copies per mL, were used to calculate the vAUCs and determine all investigation outcomes related to viral load. It is noted that applying log_{10} to one RNA copy per mL results in zero log_{10} RNA copies per mL.

Mixed model for repeated measures (MMRM)

The calculation of vAUC from Day 1 through Day 8 was performed using viral load data expressed in log_{10} RNA copies per mL, applying the linear trapezoidal rule. This method required the imputation of any missing viral load data to ensure a complete set of daily viral load measurements was available. Missing nasal swab SARS-CoV-2 viral load data were considered 'Missing at Random' and were thus imputed using a mixed-effect model for repeated measures (MMRM). The MMRM incorporated log_{10} viral load data from all visits as the dependent variable, with subject as a fixed effect and visit day as a random effect covariate. The restricted maximum likelihood method was utilized for estimation, along with the Kenward-Roger method for estimating the covariance matrix and degrees of freedom. The model employed an unstructured covariance type, which allowed for the adjustment of correlations between visit days (time points) within subjects. The treatment effect was not included as a covariate when estimating missing values. After the imputation of missing values, vAUC values were calculated.

Adjustment for baseline imbalances (ANCOVA)

Following the processing of viral load data and MMRM-based imputation, the calculated vAUC values were adjusted using analysis of covariance (ANCOVA) to control for baseline imbalances. The ANCOVA model included two fixed effect terms: the treatment group and, due to its relevance for the analyzed outcome (vAUC), the baseline (Day 1) viral load expressed as log_{10} RNA copies per mL as a continuous covariate. This method enhanced statistical power by reducing within-group error variance and adjusted for any pre-existing differences between individuals, thus providing a more accurate estimate of the treatment effect. The ANCOVA model was employed to calculate the least squares mean (LSM) for each group. The differences in LSM and their standard errors were used to construct the applicable test statistics and two-sided 95% confidence intervals (CIs). The same statistical approach was adopted to adjust FLU-PRO scores (total or domain-based) at each applicable assessment and evaluate group differences in LSM. In this case, however, the baseline (Day 1) FLU-PRO scores were used in the ANCOVA model as continuous covariates.

InFLUenza Patient-Reported Outcome Plus (FLU-PRO© Plus) symptoms questionnaire

The InFLUenza Patient-Reported Outcome Plus (FLU-PRO© Plus) symptoms questionnaire was administered daily during participation in the study. The collected results were used to derive all symptoms-related outcomes. This instrument consists of a 32-item questionnaire that assesses the severity of flu-like symptoms across six domains—nose, throat, eyes, chest/respiratory, gastrointestinal, and body/systemic. Respondents are requested to rate each symptom on a 5-point ordinal scale (0 to 4), with higher scores indicating greater severity. The responses to these items

result in a total (overall) symptoms score and domain-based symptoms scores. Additionally, two questions address the presence of the COVID-19-related symptoms "loss of smell" and "loss of taste", and a set of seven additional items assesses the return to usual health in a general sense, including the anchor question “Have you returned to your usual health today?”.

Several of the symptom-related outcomes required to determine the proportion of patients who were symptomatic or had recovered from flu-like symptoms. To do this, the responder criteria (RC) for the total and domain-based symptoms scores had to be established. The RC for the total symptoms score (TSS) was a predefined threshold used to assess if participants exhibited symptoms deviating from their normal health state. In the context of this investigation, experiencing such symptoms was attributed to COVID-19. The RC was derived by calculating the average TSS at the point where participants first reported returning to their usual health, as indicated by a “yes” response to the question, “Have you returned to your usual health today?”. This RC was then used to evaluate whether individual participants were free of COVID-19 symptoms at each relevant assessment. The RC for the domain symptoms score (DSS) mirrored that of the RC-TSS but applied to each of the six FLU-PRO domains, assessing whether participants were free from COVID-19 symptoms in the respective domain at each relevant assessment.

A participant was deemed symptomatic at an assessment if their symptoms score exceeded the RC. Conversely, if the score was below the RC, the participant was considered symptom-free. Participants without COVID-19 symptoms at an assessment were classified as asymptomatic if no previous assessments recorded a score above the RC; or recovered if they had a prior score above the RC but no subsequent assessments showing a score above the RC.

The RCs established from the pool of participants in this investigation were as follows:

- RC-TSS: 0.3338.
- RC-DSS for the Nose FLU-PRO domain: 0.4864
- RC-DSS for the Throat FLU-PRO domain: 0.2997
- RC-DSS for the Eyes FLU-PRO domain: 0.1421
- RC-DSS for the Chest/Respiratory FLU-PRO domain: 0.5249
- RC-DSS for the Gastrointestinal FLU-PRO domain: 0.1395
- RC-DSS for the Body/Systemic FLU-PRO domain: 0.2889

Supplementary tables

Table S1: Medical history

	Astodrimer (N=96)	Placebo (N=101)	Total (N=197)
Participants with medical history	60 (62.5%)	70 (69.3%)	130 (66.0%)
Vascular disorders	23 (24.0%)	22 (21.8%)	45 (22.8%)
Hypertension	21 (21.9%)	19 (18.8%)	40 (20.3%)
Aortic stenosis	0 (0.0%)	1 (1.0%)	1 (0.5%)
Arteriosclerosis	1 (1.0%)	0 (0.0%)	1 (0.5%)
Deep vein thrombosis	0 (0.0%)	1 (1.0%)	1 (0.5%)
Hypotension	1 (1.0%)	0 (0.0%)	1 (0.5%)
Raynaud's phenomenon	0 (0.0%)	1 (1.0%)	1 (0.5%)
Vasculitis	1 (1.0%)	0 (0.0%)	1 (0.5%)

	Astodrimer (N=96)	Placebo (N=101)	Total (N=197)
Metabolism and nutrition disorders	16 (16.7%)	14 (13.9%)	30 (15.2%)
Hypercholesterolaemia	5 (5.2%)	8 (7.9%)	13 (6.6%)
Type 2 diabetes mellitus	5 (5.2%)	4 (4.0%)	9 (4.6%)
Diabetes mellitus	5 (5.2%)	1 (1.0%)	6 (3.0%)
Type 1 diabetes mellitus	2 (2.1%)	1 (1.0%)	3 (1.5%)
Vitamin B12 deficiency	2 (2.1%)	1 (1.0%)	3 (1.5%)
Glucose tolerance impaired	1 (1.0%)	1 (1.0%)	2 (1.0%)
Overweight	1 (1.0%)	0 (0.0%)	1 (0.5%)
Vitamin D deficiency	0 (0.0%)	1 (1.0%)	1 (0.5%)
Neoplasms benign, malignant and unspecified	16 (16.7%)	13 (12.9%)	29 (14.7%)
Breast cancer	7 (7.3%)	3 (3.0%)	10 (5.1%)
Prostate cancer	2 (2.1%)	3 (3.0%)	5 (2.5%)
Lymphoma	2 (2.1%)	2 (2.0%)	4 (2.0%)
Bone cancer	0 (0.0%)	2 (2.0%)	2 (1.0%)
Lung neoplasm malignant	2 (2.1%)	0 (0.0%)	2 (1.0%)
Bladder cancer	0 (0.0%)	1 (1.0%)	1 (0.5%)
Brain neoplasm	1 (1.0%)	0 (0.0%)	1 (0.5%)
Breast cancer metastatic	0 (0.0%)	1 (1.0%)	1 (0.5%)
Gastrointestinal carcinoma	0 (0.0%)	1 (1.0%)	1 (0.5%)
Hepatic cancer	0 (0.0%)	1 (1.0%)	1 (0.5%)
Leukaemia	1 (1.0%)	0 (0.0%)	1 (0.5%)
Lip and/or oral cavity cancer	1 (1.0%)	0 (0.0%)	1 (0.5%)
Metastasis	1 (1.0%)	0 (0.0%)	1 (0.5%)
Neuroendocrine tumour of the lung	1 (1.0%)	0 (0.0%)	1 (0.5%)
Non-Hodgkin's lymphoma	1 (1.0%)	0 (0.0%)	1 (0.5%)
Oropharyngeal cancer	0 (0.0%)	1 (1.0%)	1 (0.5%)
Ovarian cancer	1 (1.0%)	0 (0.0%)	1 (0.5%)
Plasma cell myeloma	1 (1.0%)	0 (0.0%)	1 (0.5%)
Tongue neoplasm malignant stage unspecified	0 (0.0%)	1 (1.0%)	1 (0.5%)
Vipoma	0 (0.0%)	1 (1.0%)	1 (0.5%)
Respiratory, thoracic and mediastinal disorders	14 (14.6%)	12 (11.9%)	26 (13.2%)
Asthma	9 (9.4%)	7 (6.9%)	16 (8.1%)
Chronic obstructive pulmonary disease	2 (2.1%)	2 (2.0%)	4 (2.0%)
Sleep apnoea syndrome	2 (2.1%)	1 (1.0%)	3 (1.5%)
Bronchiectasis	0 (0.0%)	1 (1.0%)	1 (0.5%)
Dyspnoea	1 (1.0%)	0 (0.0%)	1 (0.5%)
Interstitial lung disease	0 (0.0%)	1 (1.0%)	1 (0.5%)
Lung disorder	1 (1.0%)	0 (0.0%)	1 (0.5%)
Pneumothorax	0 (0.0%)	1 (1.0%)	1 (0.5%)
Gastrointestinal disorders	9 (9.4%)	11 (10.9%)	20 (10.2%)
Gastrooesophageal reflux disease	2 (2.1%)	2 (2.0%)	4 (2.0%)
Irritable bowel syndrome	2 (2.1%)	2 (2.0%)	4 (2.0%)
Crohn's disease	1 (1.0%)	2 (2.0%)	3 (1.5%)
Barrett's oesophagus	1 (1.0%)	0 (0.0%)	1 (0.5%)
Burning mouth syndrome	0 (0.0%)	1 (1.0%)	1 (0.5%)
Coeliac disease	0 (0.0%)	1 (1.0%)	1 (0.5%)
Dyspepsia	1 (1.0%)	0 (0.0%)	1 (0.5%)

	Astodrimer (N=96)	Placebo (N=101)	Total (N=197)
Gastric haemorrhage	0 (0.0%)	1 (1.0%)	1 (0.5%)
Gastric ulcer	0 (0.0%)	1 (1.0%)	1 (0.5%)
Impaired gastric emptying	1 (1.0%)	0 (0.0%)	1 (0.5%)
Malabsorption	0 (0.0%)	1 (1.0%)	1 (0.5%)
Pancreatitis	1 (1.0%)	0 (0.0%)	1 (0.5%)
Musculoskeletal and connective tissue disorders	7 (7.3%)	11 (10.9%)	18 (9.1%)
Osteoarthritis	3 (3.1%)	2 (2.0%)	5 (2.5%)
Back pain	1 (1.0%)	3 (3.0%)	4 (2.0%)
Rheumatoid arthritis	1 (1.0%)	2 (2.0%)	3 (1.5%)
Fibromyalgia	0 (0.0%)	2 (2.0%)	2 (1.0%)
Osteoporosis	1 (1.0%)	1 (1.0%)	2 (1.0%)
Ankylosing spondylitis	1 (1.0%)	0 (0.0%)	1 (0.5%)
Arthralgia	0 (0.0%)	1 (1.0%)	1 (0.5%)
Arthritis	0 (0.0%)	1 (1.0%)	1 (0.5%)
Connective tissue disorder	0 (0.0%)	1 (1.0%)	1 (0.5%)
Foot deformity	1 (1.0%)	0 (0.0%)	1 (0.5%)
Psoriatic arthropathy	0 (0.0%)	1 (1.0%)	1 (0.5%)
Systemic lupus erythematosus	0 (0.0%)	1 (1.0%)	1 (0.5%)
Cardiac disorders	7 (7.3%)	10 (9.9%)	17 (8.6%)
Atrial fibrillation	3 (3.1%)	2 (2.0%)	5 (2.5%)
Cardiac failure	2 (2.1%)	1 (1.0%)	3 (1.5%)
Myocardial infarction	0 (0.0%)	3 (3.0%)	3 (1.5%)
Atrioventricular block	0 (0.0%)	1 (1.0%)	1 (0.5%)
Bradycardia	0 (0.0%)	1 (1.0%)	1 (0.5%)
Cardiac valve disease	0 (0.0%)	1 (1.0%)	1 (0.5%)
Myocardial ischaemia	1 (1.0%)	0 (0.0%)	1 (0.5%)
Myocarditis	1 (1.0%)	0 (0.0%)	1 (0.5%)
Postural orthostatic tachycardia syndrome	0 (0.0%)	1 (1.0%)	1 (0.5%)
Sinus bradycardia	0 (0.0%)	1 (1.0%)	1 (0.5%)
Wolff-Parkinson-White syndrome	1 (1.0%)	0 (0.0%)	1 (0.5%)
Surgical and medical procedures	10 (10.4%)	7 (6.9%)	17 (8.6%)
Heart valve replacement	1 (1.0%)	1 (1.0%)	2 (1.0%)
Hip arthroplasty	1 (1.0%)	1 (1.0%)	2 (1.0%)
Prostatectomy	0 (0.0%)	2 (2.0%)	2 (1.0%)
Thyroidectomy	1 (1.0%)	1 (1.0%)	2 (1.0%)
Vascular graft	1 (1.0%)	1 (1.0%)	2 (1.0%)
Cardiac ablation	0 (0.0%)	1 (1.0%)	1 (0.5%)
Cardiac operation	1 (1.0%)	0 (0.0%)	1 (0.5%)
Coronary artery bypass	1 (1.0%)	0 (0.0%)	1 (0.5%)
Gastric bypass	1 (1.0%)	0 (0.0%)	1 (0.5%)
Knee arthroplasty	0 (0.0%)	1 (1.0%)	1 (0.5%)
Knee operation	1 (1.0%)	0 (0.0%)	1 (0.5%)
Nephrectomy	1 (1.0%)	0 (0.0%)	1 (0.5%)
Oral surgery	1 (1.0%)	0 (0.0%)	1 (0.5%)
Pharyngeal operation	1 (1.0%)	0 (0.0%)	1 (0.5%)
Spinal operation	1 (1.0%)	0 (0.0%)	1 (0.5%)
Splenectomy	1 (1.0%)	0 (0.0%)	1 (0.5%)

	Astodrimer (N=96)	Placebo (N=101)	Total (N=197)
Tonsillectomy	0 (0.0%)	1 (1.0%)	1 (0.5%)
Transurethral prostatectomy	1 (1.0%)	0 (0.0%)	1 (0.5%)
Psychiatric disorders	8 (8.3%)	8 (7.9%)	16 (8.1%)
Depression	4 (4.2%)	4 (4.0%)	8 (4.1%)
Mixed anxiety and depressive disorder	3 (3.1%)	0 (0.0%)	3 (1.5%)
Anxiety	0 (0.0%)	2 (2.0%)	2 (1.0%)
Post-traumatic stress disorder	0 (0.0%)	2 (2.0%)	2 (1.0%)
Avoidant personality disorder	0 (0.0%)	1 (1.0%)	1 (0.5%)
Bipolar disorder	0 (0.0%)	1 (1.0%)	1 (0.5%)
Conversion disorder	0 (0.0%)	1 (1.0%)	1 (0.5%)
Learning disability	1 (1.0%)	0 (0.0%)	1 (0.5%)
Panic attack	0 (0.0%)	1 (1.0%)	1 (0.5%)
Perinatal depression	0 (0.0%)	1 (1.0%)	1 (0.5%)
Nervous system disorders	7 (7.3%)	8 (7.9%)	15 (7.6%)
Migraine	1 (1.0%)	3 (3.0%)	4 (2.0%)
Cerebrovascular accident	1 (1.0%)	2 (2.0%)	3 (1.5%)
Central nervous system lesion	1 (1.0%)	0 (0.0%)	1 (0.5%)
Dementia	1 (1.0%)	0 (0.0%)	1 (0.5%)
Epilepsy	1 (1.0%)	0 (0.0%)	1 (0.5%)
Multiple sclerosis	1 (1.0%)	0 (0.0%)	1 (0.5%)
Neuropathy peripheral	0 (0.0%)	1 (1.0%)	1 (0.5%)
Parkinson's disease	1 (1.0%)	0 (0.0%)	1 (0.5%)
Petit mal epilepsy	0 (0.0%)	1 (1.0%)	1 (0.5%)
Sciatica	0 (0.0%)	1 (1.0%)	1 (0.5%)
Endocrine disorders	4 (4.2%)	5 (5.0%)	9 (4.6%)
Hypothyroidism	4 (4.2%)	4 (4.0%)	8 (4.1%)
Diabetes insipidus	0 (0.0%)	1 (1.0%)	1 (0.5%)
Immune system disorders	6 (6.3%)	2 (2.0%)	8 (4.1%)
Seasonal allergy	5 (5.2%)	1 (1.0%)	6 (3.0%)
Autoimmune disorder	0 (0.0%)	1 (1.0%)	1 (0.5%)
Drug hypersensitivity	1 (1.0%)	0 (0.0%)	1 (0.5%)
Infections and infestations	3 (3.1%)	5 (5.0%)	8 (4.1%)
Anal abscess	1 (1.0%)	0 (0.0%)	1 (0.5%)
Appendicitis	1 (1.0%)	0 (0.0%)	1 (0.5%)
Bronchitis	0 (0.0%)	1 (1.0%)	1 (0.5%)
Infectious mononucleosis	0 (0.0%)	1 (1.0%)	1 (0.5%)
Lower respiratory tract infection	1 (1.0%)	0 (0.0%)	1 (0.5%)
Nail infection	1 (1.0%)	0 (0.0%)	1 (0.5%)
Osteomyelitis	0 (0.0%)	1 (1.0%)	1 (0.5%)
Pneumonia	0 (0.0%)	1 (1.0%)	1 (0.5%)
Post-acute COVID-19 syndrome	0 (0.0%)	1 (1.0%)	1 (0.5%)
Urinary tract infection	0 (0.0%)	1 (1.0%)	1 (0.5%)
Reproductive system and breast disorders	3 (3.1%)	4 (4.0%)	7 (3.6%)
Prostatomegaly	1 (1.0%)	2 (2.0%)	3 (1.5%)
Endometriosis	1 (1.0%)	0 (0.0%)	1 (0.5%)
Erectile dysfunction	1 (1.0%)	0 (0.0%)	1 (0.5%)
Polycystic ovaries	0 (0.0%)	1 (1.0%)	1 (0.5%)

	Astodrimer (N=96)	Placebo (N=101)	Total (N=197)
Vulvovaginal pain	0 (0.0%)	1 (1.0%)	1 (0.5%)
Congenital, familial and genetic disorders	3 (3.1%)	2 (2.0%)	5 (2.5%)
Congenital neurological disorder	0 (0.0%)	1 (1.0%)	1 (0.5%)
Ehlers-Danlos syndrome	0 (0.0%)	1 (1.0%)	1 (0.5%)
Hereditary haemochromatosis	1 (1.0%)	0 (0.0%)	1 (0.5%)
Huntington's disease	1 (1.0%)	0 (0.0%)	1 (0.5%)
Type IIa hyperlipidaemia	1 (1.0%)	0 (0.0%)	1 (0.5%)
Renal and urinary disorders	2 (2.1%)	3 (3.0%)	5 (2.5%)
Chronic kidney disease	1 (1.0%)	1 (1.0%)	2 (1.0%)
Hypertonic bladder	0 (0.0%)	1 (1.0%)	1 (0.5%)
Renal failure	1 (1.0%)	0 (0.0%)	1 (0.5%)
Urinary retention	0 (0.0%)	1 (1.0%)	1 (0.5%)
Eye disorders	1 (1.0%)	2 (2.0%)	3 (1.5%)
Glaucoma	1 (1.0%)	2 (2.0%)	3 (1.5%)
Skin and subcutaneous tissue disorders	2 (2.1%)	1 (1.0%)	3 (1.5%)
Dermatomyositis	1 (1.0%)	0 (0.0%)	1 (0.5%)
Eczema	1 (1.0%)	0 (0.0%)	1 (0.5%)
Psoriasis	0 (0.0%)	1 (1.0%)	1 (0.5%)
Social circumstances	2 (2.1%)	1 (1.0%)	3 (1.5%)
Vascular device user	1 (1.0%)	1 (1.0%)	2 (1.0%)
Menopause	1 (1.0%)	0 (0.0%)	1 (0.5%)
Blood and lymphatic system disorders	0 (0.0%)	2 (2.0%)	2 (1.0%)
Anaemia	0 (0.0%)	1 (1.0%)	1 (0.5%)
Mast cell activation syndrome	0 (0.0%)	1 (1.0%)	1 (0.5%)
Investigations	2 (2.1%)	0 (0.0%)	2 (1.0%)
Heart rate decreased	1 (1.0%)	0 (0.0%)	1 (0.5%)
White blood cell count decreased	1 (1.0%)	0 (0.0%)	1 (0.5%)
Ear and labyrinth disorders	0 (0.0%)	1 (1.0%)	1 (0.5%)
Vertigo	0 (0.0%)	1 (1.0%)	1 (0.5%)
Injury, poisoning and procedural complications	1 (1.0%)	0 (0.0%)	1 (0.5%)
Traumatic arthropathy	1 (1.0%)	0 (0.0%)	1 (0.5%)
Pregnancy, puerperium and perinatal conditions	0 (0.0%)	1 (1.0%)	1 (0.5%)
Gestational hypertension	0 (0.0%)	1 (1.0%)	1 (0.5%)

Data are n (%). Verbatim terms coded using MedDRA version 26.1

Table S2: Peak nasal swab SARS-CoV-2 RNA load post-baseline

	Astodrimer	Placebo	Astodrimer versus placebo
All ages (mITT)			
N	96	101	
LSM (SE)	5.6 (0.1)	5.7 (0.1)	-0.1 (0.2)
95% CI	5.3, 5.8	5.5, 6.0	-0.5, 0.2
p-value			0.4578
Age 40Y+			
N	68	75	
LSM (SE)	5.5 (0.1)	5.8 (0.1)	-0.2 (0.2)
95% CI	5.3, 5.8	5.5, 6.1	-0.6, 0.2
p-value			0.234
Age 45Y+			
N	56	62	
LSM (SE)	5.5 (0.2)	6.0 (0.2)	-0.5 (0.2)
95% CI	5.2, 5.8	5.7, 6.3	-0.9, 0.0
p-value			0.030*
Age 50Y+			
N	49	52	
LSM (SE)	5.6 (0.2)	6.0 (0.2)	-0.4 (0.2)
95% CI	5.3, 5.9	5.7, 6.4	-0.9, 0.0
p-value			0.059
Age 55Y+			
N	39	45	
LSM (SE)	5.5 (0.2)	6.1 (0.2)	-0.6 (0.3)
95% CI	5.1, 5.9	5.8, 6.5	-1.1, -0.1
p-value			0.022*
Age 60Y+			
N	30	36	
LSM (SE)	5.3 (0.2)	6.3 (0.2)	-1.0 (0.3)
95% CI	4.9, 5.8	5.9, 6.7	-1.5, -0.4
p-value			0.002*
Age 65Y+			
N	23	27	
LSM (SE)	5.2 (0.3)	6.4 (0.3)	-1.2 (0.4)
95% CI	4.7, 5.8	5.9, 6.9	-1.9, -0.4
p-value			0.003*

Values expressed as log_{10} SARS-CoV-2 RNA copies per mL.

LSM=Least squares mean; SE=Standard error; CI=Confidence interval; *p<0.05.

The LSM results from the application of an ANCOVA model with two fixed effect terms: treatment group and the log_{10} of viral load at Day 1 (baseline) as continuous covariate.

Table S3: Time in days from Day 1 to peak nasal swab SARS-CoV-2 RNA load post-baseline

	Astodrimer	Placebo	Astodrimer versus placebo
All ages (mITT)			
N	96	101	
Mean (SD)	1.5 (0.1)	1.6 (0.1)	-0.1 (0.1)
95% CI	1.3, 1.7	1.4, 1.8	-0.4, 0.2
p-value			0.449
Age 40Y+			
N	68	75	
Mean (SD)	1.5 (0.1)	1.7 (0.1)	-0.2 (0.2)
95% CI	1.2, 1.7	1.5, 1.9	-0.5, 0.1
p-value			0.165
Age 45Y+			
N	56	62	
Mean (SD)	1.5 (0.1)	1.7 (0.1)	-0.2 (0.2)
95% CI	1.2, 1.8	1.5, 2.0	-0.6, 0.1
p-value			0.203
Age 50Y+			
N	49	52	
Mean (SD)	1.5 (0.1)	1.8 (0.1)	-0.3 (0.2)
95% CI	1.3, 1.8	1.5, 2.1	-0.7, 0.1
p-value			0.170
Age 55Y+			
N	39	45	
Mean (SD)	1.6 (0.2)	1.9 (0.2)	-0.3 (0.2)
95% CI	1.2, 1.9	1.6, 2.3	-0.8, 0.1
p-value			0.172
Age 60Y+			
N	30	36	
Mean (SD)	1.6 (0.2)	2.0 (0.2)	-0.4 (0.3)
95% CI	1.2, 2.0	1.6, 2.4	-1.0, 0.1
p-value			0.140
Age 65Y+			
N	23	27	
Mean (SD)	1.5 (0.2)	1.9 (0.2)	-0.4 (0.3)
95% CI	1.1, 1.9	1.5, 2.2	-0.9, 0.2
p-value			0.216

SD=Standard deviation; CI=Confidence interval.

Table S4: Nasal swab SARS-CoV-2 RNA load at each determination

	Baseline (Day 1)	Day 2	Day 3	Day 4	Day 5	Day 6	Day 7	Day 8
All ages (mITT)								
Astodrimer [LSM (SE)]	5.6 (0.2)	5.3 (0.1)	4.8 (0.1)	4.1 (0.2)	3.4 (0.2)	2.7 (0.2)	2.4 (0.2)	2.0 (0.2)
Placebo [LSM (SE)]	5.5 (0.2)	5.2 (0.1)	4.8 (0.1)	4.3 (0.2)	3.7 (0.2)	3.1 (0.2)	2.5 (0.2)	2.2 (0.2)
Viral load reduction	NA	-20.6%	0.0%	36.9%	49.9%	60.2%	20.6%	36.9%
p-value	NA	0.670	0.674	0.425	0.260	0.126	0.559	0.381
Age 40Y+								
Astodrimer [LSM (SE)]	5.6 (0.2)	5.3 (0.1)	4.7 (0.2)	4.0 (0.2)	3.4 (0.2)	2.9 (0.2)	2.6 (0.2)	2.1 (0.2)
Placebo [LSM (SE)]	5.6 (0.2)	5.3 (0.1)	4.9 (0.2)	4.4 (0.2)	3.9 (0.2)	3.4 (0.2)	2.8 (0.2)	2.4 (0.2)
Viral load reduction	NA	0.0%	36.9%	60.2%	68.4%	68.4%	36.9%	49.9%
p-value	NA	0.861	0.352	0.132	0.118	0.067	0.517	0.274
Age 45Y+								
Astodrimer [LSM (SE)]	5.6 (0.2)	5.3 (0.1)	4.8 (0.2)	4.1 (0.2)	3.4 (0.2)	2.9 (0.2)	2.6 (0.2)	2.2 (0.2)
Placebo [LSM (SE)]	5.7 (0.2)	5.5 (0.1)	5.2 (0.2)	4.7 (0.2)	4.2 (0.2)	3.8 (0.2)	3.2 (0.2)	2.7 (0.2)
Viral load reduction	NA	36.9%	60.2%	74.9%	84.2%	87.4%	74.9%	68.4%
p-value	NA	0.195	0.125	0.043*	0.012*	0.008*	0.089	0.083
Age 50Y+								
Astodrimer [LSM (SE)]	5.7 (0.2)	5.4 (0.1)	5.0 (0.2)	4.2 (0.2)	3.5 (0.2)	3.0 (0.2)	2.8 (0.2)	2.3 (0.2)
Placebo [LSM (SE)]	5.8 (0.2)	5.5 (0.1)	5.3 (0.2)	4.8 (0.2)	4.4 (0.2)	4.0 (0.2)	3.4 (0.2)	2.9 (0.2)
Viral load reduction	NA	20.6%	49.9%	74.9%	87.4%	90.0%	74.9%	74.9%
p-value	NA	0.344	0.307	0.064	0.009*	0.005*	0.068	0.084
Age 55Y+								
Astodrimer [LSM (SE)]	5.5 (0.2)	5.3 (0.1)	4.9 (0.2)	4.2 (0.2)	3.7 (0.3)	3.1 (0.3)	2.8 (0.3)	2.2 (0.3)
Placebo [LSM (SE)]	5.8 (0.2)	5.5 (0.1)	5.3 (0.2)	4.8 (0.2)	4.4 (0.3)	4.1 (0.3)	3.5 (0.3)	3.0 (0.3)
Viral load reduction	NA	36.9%	60.2%	74.9%	80.0%	90.0%	80.0%	84.2%
p-value	NA	0.307	0.157	0.053	0.051	0.010*	0.086	0.047*
Age 60Y+								
Astodrimer [LSM (SE)]	5.4 (0.3)	5.1 (0.2)	4.8 (0.2)	4.1 (0.3)	3.6 (0.3)	2.9 (0.3)	2.6 (0.3)	1.9 (0.3)
Placebo [LSM (SE)]	5.8 (0.3)	5.6 (0.2)	5.4 (0.2)	5.0 (0.3)	4.6 (0.3)	4.4 (0.3)	3.7 (0.3)	3.1 (0.3)
Viral load reduction	NA	68.4%	74.9%	87.4%	90.0%	96.8%	92.1%	93.7%
p-value	NA	0.065	0.019*	0.021*	0.018*	0.002*	0.025*	0.011*
Age 65Y+								
Astodrimer [LSM (SE)]	5.6 (0.3)	5.0 (0.2)	4.7 (0.2)	3.9 (0.3)	3.6 (0.3)	3.0 (0.4)	2.8 (0.4)	2.1 (0.4)
Placebo [LSM (SE)]	5.7 (0.3)	5.7 (0.2)	5.5 (0.2)	5.2 (0.3)	5.0 (0.3)	4.5 (0.4)	3.8 (0.4)	3.3 (0.4)
Viral load reduction	NA	80.0%	84.2%	95.0%	96.0%	96.8%	90.0%	93.7%
p-value	NA	0.008*	0.013*	0.006*	0.008*	0.015*	0.062	0.048*

Values expressed as log_{10} SARS-CoV-2 RNA copies per mL.

The percent reductions in viral load indicate the change in the astodrimer group relative to the placebo group. Positive reductions denote a lower viral load in the astodrimer group compared to the placebo group. Conversely, a negative reduction indicates a higher viral load in the astodrimer group than in the placebo group.

LSM=Least squares mean; SE=Standard error; CI=Confidence interval; *p<0.05.

The LSM results from the application of an ANCOVA model with two fixed effect terms: treatment group and the log_{10} of viral load at Day 1 (baseline) as continuous covariate.

Table S5: Mean change in nasal swab SARS-CoV-2 RNA load at each determination relative to baseline (Day 1)

	Day 2	Day 3	Day 4	Day 5	Day 6	Day 7	Day 8
All ages (mITT)							
Astodrimer [LSM (VLR)]	-0.3 (49.881%)	-0.8 (84.151%)	-1.5 (96.838%)	-2.2 (99.369%)	-2.9 (99.874%)	-3.2 (99.937%)	-3.6 (99.975%)
Placebo [LSM (VLR)]	-0.3 (49.881%)	-0.7 (80.047%)	-1.3 (94.988%)	-1.9 (98.741%)	-2.5 (99.684%)	-3.1 (99.921%)	-3.4 (99.960%)
p-value	0.670	0.674	0.425	0.260	0.126	0.559	0.381
Age 40Y+							
Astodrimer [LSM (VLR)]	-0.3 (49.881%)	-0.9 (87.411%)	-1.6 (97.488%)	-2.2 (99.369%)	-2.7 (99.800%)	-3.0 (99.900%)	-3.5 (99.968%)
Placebo [LSM (VLR)]	-0.3 (49.881%)	-0.7 (80.047%)	-1.2 (93.690%)	-1.7 (98.005%)	-2.2 (99.369%)	-2.8 (99.842%)	-3.2 (99.937%)
p-value	0.861	0.352	0.132	0.118	0.067	0.517	0.274
Age 45Y+							
Astodrimer [LSM (VLR)]	-0.4 (60.189%)	-0.9 (87.411%)	-1.6 (97.488%)	-2.2 (99.369%)	-2.8 (99.842%)	-3.0 (99.900%)	-3.5 (99.968%)
Placebo [LSM (VLR)]	-0.2 (36.904%)	-0.5 (68.377%)	-1.0 (90.000%)	-1.4 (96.019%)	-1.9 (98.741%)	-2.5 (99.684%)	-3.0 (99.900%)
p-value	0.195	0.125	0.043*	0.012*	0.008*	0.089	0.083
Age 50Y+							
Astodrimer [LSM (VLR)]	-0.4 (60.189%)	-0.7 (80.047%)	-1.5 (96.838%)	-2.2 (99.369%)	-2.7 (99.800%)	-2.9 (99.874%)	-3.4 (99.960%)
Placebo [LSM (VLR)]	-0.2 (36.904%)	-0.5 (68.377%)	-0.9 (87.411%)	-1.3 (94.988%)	-1.7 (98.005%)	-2.3 (99.499%)	-2.8 (99.842%)
p-value	0.344	0.307	0.064	0.009*	0.005*	0.068	0.084
Age 55Y+							
Astodrimer [LSM (VLR)]	-0.4 (60.189%)	-0.8 (84.151%)	-1.5 (96.838%)	-2.0 (99.000%)	-2.6 (99.749%)	-2.9 (99.874%)	-3.5 (99.968%)
Placebo [LSM (VLR)]	-0.2 (36.904%)	-0.4 (60.189%)	-0.8 (84.151%)	-1.3 (94.988%)	-1.5 (96.838%)	-2.2 (99.369%)	-2.7 (99.800%)
p-value	0.307	0.157	0.053	0.051	0.010*	0.086	0.047*
Age 60Y+							
Astodrimer [LSM (VLR)]	-0.5 (68.377%)	-0.9 (87.411%)	-1.5 (96.838%)	-2.0 (99.000%)	-2.8 (99.842%)	-3.0 (99.900%)	-3.7 (99.980%)
Placebo [LSM (VLR)]	-0.1 (20.567%)	-0.2 (36.904%)	-0.6 (74.881%)	-1.0 (90.000%)	-1.2 (93.690%)	-1.9 (98.741%)	-2.5 (99.684%)
p-value	0.065	0.019*	0.021*	0.018*	0.002*	0.025*	0.011*

	Day 2	Day 3	Day 4	Day 5	Day 6	Day 7	Day 8
Age 65Y+							
Astodrimer [LSM (VLR)]	-0.7 (80.047%)	-1.0 (90.000%)	-1.8 (98.415%)	-2.1 (99.206%)	-2.7 (99.800%)	-2.9 (99.874%)	-3.6 (99.975%)
Placebo [LSM (VLR)]	0.0 (0.000%)	-0.2 (36.904%)	-0.5 (68.377%)	-0.7 (80.047%)	-1.2 (93.690%)	-1.8 (98.415%)	-2.4 (99.602%)
p-value	0.008*	0.013*	0.006*	0.008*	0.015*	0.062	0.048*

LSM=Least squares mean; VLR=Viral load reduction; * $p<0.05$.

The LSM results from the application of an ANCOVA model with two fixed effect terms: treatment group and the $\log_{10}$ of viral load at Day 1 (baseline) as continuous covariate.

Viral load change expressed as $\log_{10}$ RNA copies per mL and percent reduction relative to the Day 1 (baseline) viral load determination.

p-values refer to the difference in mean viral load change ($\log_{10}$ RNA copies per mL) between astodrimer and placebo at each day.

Table S6: Proportion of symptomatic and recovered participants as per the throat symptoms score

	Day 1 (BL)	Day 2		Day 3		Day 4		Day 5		Day 6		Day 7		Day 8	
	Symp	Symp	Rec	Symp	Rec	Symp	Rec	Symp	Rec	Symp	Rec	Symp	Rec	Symp	Rec
All ages (mITT)															
Astodrimer	89.4%	82.2%	13.3%	72.4%	23.0%	63.0%	32.6%	49.4%	46.1%	40.0%	55.6%	30.3%	65.2%	18.6%	77.9%
Placebo	87.0%	74.7%	16.2%	65.3%	25.5%	57.7%	33.0%	48.0%	42.9%	37.5%	54.2%	30.9%	59.6%	23.7%	67.0%
p-value			0.352		0.429		0.415		0.491		0.597		0.426		0.176
Age 40Y+															
Astodrimer	89.4%	81.0%	14.3%	66.7%	28.3%	60.9%	34.4%	53.2%	41.9%	43.5%	51.6%	31.1%	63.9%	18.3%	78.3%
Placebo	90.5%	75.3%	16.4%	67.6%	24.3%	60.3%	31.5%	50.7%	41.1%	38.0%	53.5%	31.0%	60.6%	26.4%	65.3%
p-value			0.702		0.712		0.702		0.796		0.652		0.816		0.236
Age 45Y+															
Astodrimer	89.1%	78.4%	15.7%	65.3%	28.6%	57.7%	36.5%	52.9%	41.2%	46.0%	48.0%	34.7%	59.2%	20.4%	75.5%
Placebo	91.8%	80.0%	13.3%	72.1%	21.3%	66.7%	26.7%	56.7%	36.7%	44.8%	48.3%	37.9%	55.2%	32.2%	61.0%
p-value			0.937		0.690		0.521		0.908		1.000		0.951		0.280
Age 50Y+															
Astodrimer	87.5%	75.0%	18.2%	64.3%	28.6%	60.0%	33.3%	54.5%	38.6%	48.8%	44.2%	35.7%	57.1%	19.0%	76.2%
Placebo	92.2%	80.0%	12.0%	72.5%	19.6%	68.0%	24.0%	60.0%	32.0%	46.9%	44.9%	39.6%	52.1%	36.7%	55.1%
p-value			0.755		0.585		0.606		0.853		1.000		0.947		0.107
Age 55Y+															
Astodrimer	86.8%	74.3%	17.1%	60.6%	30.3%	58.3%	33.3%	51.4%	40.0%	47.1%	44.1%	32.4%	58.8%	15.2%	78.8%
Placebo	90.9%	81.4%	9.3%	75.0%	15.9%	72.1%	18.6%	65.1%	25.6%	52.4%	38.1%	42.9%	47.6%	39.5%	51.2%
p-value			0.563		0.376		0.353		0.420		0.888		0.587		0.033*
Age 60Y+															
Astodrimer	83.3%	70.4%	18.5%	54.2%	33.3%	59.3%	29.6%	53.8%	34.6%	52.0%	36.0%	36.0%	52.0%	12.5%	79.2%
Placebo	91.4%	85.3%	5.9%	80.0%	11.4%	79.4%	11.8%	70.6%	20.6%	57.6%	33.3%	45.5%	45.5%	41.2%	50.0%
p-value			0.278		0.078		0.167		0.360		0.864		0.742		0.038*
Age 65Y+															
Astodrimer	78.3%	65.0%	20.0%	47.4%	36.8%	50.0%	35.0%	52.6%	31.6%	50.0%	33.3%	33.3%	50.0%	11.8%	76.5%
Placebo	88.5%	80.8%	7.7%	76.9%	11.5%	80.0%	8.0%	76.0%	12.0%	58.3%	29.2%	45.8%	41.7%	40.0%	48.0%
p-value			0.502		0.094		0.066		0.182		0.834		0.694		0.123

Symp=Symptomatic for COVID-19 symptoms; Rec=Recovered from COVID-19 symptoms; p-value refers to the difference in the proportion of symptomatic and recovered participants. * $p<0.05$.

Table S7: Proportion of symptomatic and recovered participants as per the eyes symptoms score

	Day 1 (BL)	Day 2		Day 3		Day 4		Day 5		Day 6		Day 7		Day 8	
	Symp	Symp	Rec	Symp	Rec	Symp	Rec	Symp	Rec	Symp	Rec	Symp	Rec	Symp	Rec
All ages (mITT)															
Astodrimer	70.2%	63.3%	16.7%	50.6%	33.3%	42.4%	41.3%	34.8%	48.3%	27.8%	56.7%	20.2%	65.2%	12.8%	70.9%
Placebo	67.0%	60.6%	10.1%	52.0%	20.4%	50.5%	23.7%	36.7%	38.8%	33.3%	41.7%	23.4%	53.2%	18.6%	60.8%
p-value			0.208		0.060		0.030*		0.329		0.095		0.209		0.362
Age 40Y+															
Astodrimer	65.2%	58.7%	15.9%	50.0%	30.0%	39.1%	40.6%	30.6%	48.4%	24.2%	56.5%	18.0%	63.9%	13.3%	66.7%
Placebo	68.9%	65.8%	6.8%	59.5%	16.2%	57.5%	19.2%	41.1%	37.0%	35.2%	42.3%	26.8%	53.5%	20.8%	59.7%
p-value			0.264		0.171		0.020*		0.374		0.252		0.405		0.557
Age 45Y+															
Astodrimer	61.8%	56.9%	15.7%	49.0%	30.6%	40.4%	38.5%	31.4%	47.1%	26.0%	54.0%	18.4%	63.3%	14.3%	65.3%
Placebo	68.9%	66.7%	6.7%	62.3%	13.1%	60.0%	16.7%	43.3%	35.0%	37.9%	39.7%	29.3%	51.7%	23.7%	57.6%
p-value			0.298		0.089		0.025*		0.382		0.328		0.415		0.505
Age 50Y+															
Astodrimer	64.6%	59.1%	15.9%	50.0%	31.0%	40.0%	40.0%	31.8%	47.7%	25.6%	55.8%	21.4%	61.9%	16.7%	64.3%
Placebo	70.6%	66.0%	8.0%	62.7%	11.8%	64.0%	12.0%	48.0%	30.0%	40.8%	36.7%	31.3%	50.0%	26.5%	55.1%
p-value			0.508		0.076		0.006*		0.170		0.182		0.503		0.564
Age 55Y+															
Astodrimer	63.2%	60.0%	17.1%	51.5%	30.3%	44.4%	36.1%	34.3%	45.7%	26.5%	55.9%	20.6%	61.8%	18.2%	63.6%
Placebo	72.7%	67.4%	9.3%	63.6%	13.6%	65.1%	11.6%	46.5%	30.2%	42.9%	33.3%	33.3%	45.2%	30.2%	51.2%
p-value			0.633		0.234		0.035*		0.413		0.144		0.343		0.466
Age 60Y+															
Astodrimer	63.3%	55.6%	18.5%	41.7%	37.5%	37.0%	40.7%	26.9%	50.0%	20.0%	60.0%	12.0%	68.0%	12.5%	66.7%
Placebo	74.3%	70.6%	8.8%	65.7%	14.3%	67.6%	11.8%	52.9%	26.5%	51.5%	27.3%	39.4%	42.4%	32.4%	50.0%
p-value			0.417		0.088		0.020*		0.102		0.022*		0.062		0.273
Age 65Y+															
Astodrimer	56.5%	55.0%	15.0%	42.1%	31.6%	35.0%	35.0%	31.6%	36.8%	22.2%	50.0%	11.1%	61.1%	11.8%	58.8%
Placebo	73.1%	73.1%	7.7%	69.2%	11.5%	72.0%	8.0%	52.0%	28.0%	54.2%	25.0%	37.5%	45.8%	32.0%	52.0%
p-value			0.402		0.156		0.027*		0.435		0.100		0.154		0.310

Symp=Symptomatic for COVID-19 symptoms; Rec=Recovered from COVID-19 symptoms; p-value refers to the difference in the proportion of symptomatic and recovered participants. * $p<0.05$.

Table S8: Proportion of symptomatic participants who recovered from COVID-19 symptoms at or by Day 8 as per the total and domain-based symptoms scores

	Total	Nose	Throat	Eyes	Chest/respiratory	Gastrointestinal	Body/systemic
All ages (mITT)							
Astodrimer [Events/N (%)]	56/79 (70.9)	52/81 (64.2)	55/71 (77.5)	45/56 (80.4)	44/72 (61.1)	47/60 (78.3)	56/79 (70.9)
Placebo [Events/N (%)]	55/86 (64.0)	55/91 (60.4)	50/73 (68.5)	49/67 (73.1)	38/78 (48.7)	44/60 (73.3)	55/83 (66.3)
Rate Difference (%)	6.9	3.8	9.0	7.3	12.4	5.0	4.6
p-value	0.407	0.639	0.263	0.398	0.142	0.670	0.612
Age 40Y+							
Astodrimer [Events/N (%)]	39/55 (70.9)	34/55 (61.8)	38/49 (77.6)	29/37 (78.4)	28/49 (57.1)	31/41 (75.6)	39/55 (70.9)
Placebo [Events/N (%)]	40/63 (63.5)	41/68 (60.3)	36/55 (65.5)	38/53 (71.7)	28/56 (50.0)	34/42 (81.0)	40/62 (64.5)
Rate Difference (%)	7.4	1.5	12.1	6.7	7.1	-5.4	6.4
p-value	0.437	1.000	0.199	0.624	0.557	0.603	0.554
Age 45Y+							
Astodrimer [Events/N (%)]	30/45 (66.7)	26/45 (57.8)	29/39 (74.4)	23/30 (76.7)	21/40 (52.5)	25/32 (78.1)	31/45 (68.9)
Placebo [Events/N (%)]	30/52 (57.7)	32/56 (57.1)	29/48 (60.4)	30/44 (68.2)	23/48 (47.9)	26/34 (76.5)	30/51 (58.8)
Rate Difference (%)	9.0	0.7	14.0	8.5	4.6	1.6	10.1
p-value	0.407	1.000	0.253	0.600	0.831	1.000	0.396
Age 50Y+							
Astodrimer [Events/N (%)]	23/38 (60.5)	21/39 (53.8)	24/32 (75.0)	19/26 (73.1)	15/33 (45.5)	21/26 (80.8)	24/38 (63.2)
Placebo [Events/N (%)]	24/43 (55.8)	25/46 (54.3)	22/40 (55.0)	23/36 (63.9)	17/40 (42.5)	20/26 (76.9)	24/43 (55.8)
Rate Difference (%)	4.7	-0.5	20.0	9.2	3.0	3.9	7.4
p-value	0.822	1.000	0.090	0.584	0.817	1.000	0.651
Age 55Y+							
Astodrimer [Events/N (%)]	17/29 (58.6)	14/30 (46.7)	20/25 (80.0)	14/20 (70.0)	10/25 (40.0)	15/19 (78.9)	18/29 (62.1)
Placebo [Events/N (%)]	19/37 (51.4)	21/40 (52.5)	18/35 (51.4)	18/31 (58.1)	13/35 (37.1)	19/25 (76.0)	19/37 (51.4)
Rate Difference (%)	7.2	-5.8	28.6	11.9	2.9	2.9	10.7
p-value	0.623	0.809	0.031*	0.554	1.000	1.000	0.458
Age 60Y+							
Astodrimer [Events/N (%)]	12/20 (60.0)	11/21 (52.4)	14/17 (82.4)	10/13 (76.9)	9/19 (47.4)	11/14 (78.6)	13/20 (65.0)
Placebo [Events/N (%)]	16/30 (53.3)	16/32 (50.0)	15/29 (51.7)	14/25 (56.0)	9/29 (31.0)	15/20 (75.0)	15/29 (51.7)
Rate Difference (%)	6.7	2.4	30.7	20.9	16.4	3.6	13.3
p-value	0.773	1.000	0.058	0.294	0.362	1.000	0.394

	Total	Nose	Throat	Eyes	Chest/respiratory	Gastrointestinal	Body/systemic
Age 65Y+							
Astodrimer [Events/N (%)]	7/13 (53.8)	7/14 (50.0)	9/11 (81.8)	6/8 (75.0)	4/12 (33.3)	9/10 (90.0)	9/13 (69.2)
Placebo [Events/N (%)]	10/21 (47.6)	10/23 (43.5)	11/21 (52.4)	11/19 (57.9)	5/21 (23.8)	11/15 (73.3)	8/21 (38.1)
Rate Difference (%)	6.2	6.5	29.4	17.1	9.5	16.7	31.1
p-value	1.000	0.745	0.139	0.665	0.690	0.615	0.157

* p<0.05.

Table S9: In participants reporting COVID-19 symptoms as per the total or domain-based symptom scores, time in days from Day 1 to recovery from symptoms

	Total	Nose	Throat	Eyes	Chest/respiratory	Gastrointestinal	Body/systemic
All ages (mITT)							
Astodrimer [Events/N (%)]	62/93 (66.7)	55/93 (59.1)	69/91 (75.8)	62/79 (78.5)	56/90 (62.2)	58/75 (77.3)	62/92 (67.4)
Placebo [Events/N (%)]	65/98 (66.3)	60/97 (61.9)	69/92 (75.0)	61/80 (76.2)	50/91 (54.9)	53/71 (74.6)	66/97 (68.0)
Rate Difference (%)	0.3	-2.7	0.8	2.2	7.3	2.7	-0.6
p-value (log rank)	0.879	0.903	0.866	0.131	0.235	0.567	0.914
Hazard Ratio	0.98	0.98	1.03	1.28	1.24	1.10	0.98
p-value (cox)	0.889	0.910	0.879	0.175	0.265	0.605	0.921
Age 40Y+							
Astodrimer [Events/N (%)]	44/66 (66.7)	37/65 (56.9)	48/64 (75.0)	41/53 (77.4)	37/63 (58.7)	39/52 (75.0)	42/64 (65.6)
Placebo [Events/N (%)]	47/72 (65.3)	44/72 (61.1)	50/69 (72.5)	45/61 (73.8)	38/67 (56.7)	40/50 (80.0)	47/71 (66.2)
Rate Difference (%)	1.4	-4.2	2.5	3.6	2.0	-5.0	-0.6
p-value (log rank)	0.963	0.984	0.710	0.072	0.559	0.728	0.906
Hazard Ratio	1.01	1.00	1.07	1.42	1.14	0.93	0.98
p-value (cox)	0.966	0.985	0.735	0.104	0.583	0.754	0.913
Age 45Y+							
Astodrimer [Events/N (%)]	34/54 (63.0)	29/54 (53.7)	38/52 (73.1)	33/43 (76.7)	27/51 (52.9)	32/42 (76.2)	33/52 (63.5)
Placebo [Events/N (%)]	36/60 (60.0)	35/60 (58.3)	39/58 (67.2)	36/51 (70.6)	31/57 (54.4)	30/40 (75.0)	35/58 (60.3)
Rate Difference (%)	3.0	-4.6	5.8	6.2	-1.4	1.2	3.1
p-value (log rank)	0.797	0.961	0.380	0.063	0.796	0.673	0.839
Hazard Ratio	1.06	1.01	1.20	1.50	1.07	1.10	1.05
p-value (cox)	0.811	0.964	0.420	0.092	0.806	0.703	0.850
Age 50Y+							
Astodrimer [Events/N (%)]	27/47 (57.4)	23/47 (48.9)	33/45 (73.3)	28/38 (73.7)	21/44 (47.7)	27/35 (77.1)	26/45 (57.8)
Placebo [Events/N (%)]	30/51 (58.8)	28/50 (56.0)	30/48 (62.5)	29/43 (67.4)	25/49 (51.0)	24/32 (75.0)	28/49 (57.1)
Rate Difference (%)	-1.4	-7.1	10.8	6.2	-3.3	2.1	0.6
p-value (log rank)	0.864	0.896	0.229	0.061	0.997	0.583	0.951
Hazard Ratio	0.96	0.97	1.32	1.58	1.00	1.15	1.02
p-value (cox)	0.873	0.902	0.269	0.087	0.997	0.622	0.954

	Total	Nose	Throat	Eyes	Chest/respiratory	Gastrointestinal	Body/systemic
Age 55Y+							
Astodrimer [Events/N (%)]	21/37 (56.8)	16/37 (43.2)	27/35 (77.1)	22/30 (73.3)	15/34 (44.1)	20/26 (76.9)	20/35 (57.1)
Placebo [Events/N (%)]	25/44 (56.8)	24/43 (55.8)	24/41 (58.5)	24/37 (64.9)	21/43 (48.8)	21/28 (75.0)	23/42 (54.8)
Rate Difference (%)	-0.1	-12.6	18.6	8.5	-4.7	1.9	2.4
p-value (log rank)	0.996	0.618	0.061	0.092	0.841	0.363	0.942
Hazard Ratio	1.00	0.86	1.62	1.58	0.94	1.29	1.02
p-value (cox)	0.996	0.636	0.086	0.124	0.851	0.410	0.946
Age 60Y+							
Astodrimer [Events/N (%)]	16/28 (57.1)	13/28 (46.4)	20/26 (76.9)	17/22 (77.3)	13/27 (48.1)	13/18 (72.2)	15/26 (57.7)
Placebo [Events/N (%)]	20/35 (57.1)	18/34 (52.9)	19/33 (57.6)	19/30 (63.3)	15/35 (42.9)	17/23 (73.9)	18/33 (54.5)
Rate Difference (%)	0.0	-6.5	19.3	13.9	5.3	-1.7	3.1
p-value (log rank)	0.924	0.819	0.075	0.020*	0.591	0.781	0.931
Hazard Ratio	1.03	1.08	1.68	2.05	1.21	1.10	1.03
p-value (cox)	0.928	0.828	0.106	0.035*	0.612	0.801	0.936
Age 65Y+							
Astodrimer [Events/N (%)]	11/21 (52.4)	9/21 (42.9)	14/19 (73.7)	11/15 (73.3)	8/20 (40.0)	10/13 (76.9)	11/19 (57.9)
Placebo [Events/N (%)]	14/26 (53.8)	12/25 (48.0)	14/24 (58.3)	15/23 (65.2)	10/26 (38.5)	13/18 (72.2)	11/25 (44.0)
Rate Difference (%)	-1.5	-5.1	15.4	8.1	1.5	4.7	13.9
p-value (log rank)	0.715	0.783	0.115	0.085	0.688	0.325	0.270
Hazard Ratio	1.15	1.12	1.71	1.88	1.20	1.45	1.56
p-value (cox)	0.733	0.791	0.156	0.119	0.703	0.378	0.301

* $p<0.05$.

Table S10: Proportion of participants reporting loss of sense of smell and/or taste on any one day after starting with the study treatment

	Smell	Taste	Smell or Taste
All ages (mITT)			
Astodrimer [Events/N (%)]	35/94 (37.2%)	41/94 (43.6)	47/94 (50.0)
Placebo [Events/N (%)]	50/101 (49.5%)	50/101 (49.5)	59/101 (58.4)
Rate Difference (%)	-12.3	-5.9	-8.4
p-value	0.1117	0.473	0.253
Age 40Y+			
Astodrimer [Events/N (%)]	24/66 (36.4)	27/66 (40.9)	31/66 (47.0)
Placebo [Events/N (%)]	37/75 (49.3)	39/75 (52.0)	45/75 (60.0)
Rate Difference (%)	-13.0	-11.1	-13.0
p-value	0.129	0.237	0.131
Age 45Y+			
Astodrimer [Events/N (%)]	18/54 (33.3)	22/54 (40.7)	24/54 (44.4)
Placebo [Events/N (%)]	32/62 (51.6)	33/62 (53.2)	39/62 (62.9)
Rate Difference (%)	-18.3	-12.5	-18.5
p-value	0.061	0.197	0.062
Age 50Y+			
Astodrimer [Events/N (%)]	14/47 (29.8)	18/47 (38.3)	19/47 (40.4)
Placebo [Events/N (%)]	26/52 (50.0)	28/52 (53.8)	33/52 (63.5)
Rate Difference (%)	-20.2	-15.5	-23.0
p-value	0.064	0.158	0.027*
Age 55Y+			
Astodrimer [Events/N (%)]	11/38 (28.9)	16/38 (42.1)	16/38 (42.1)
Placebo [Events/N (%)]	23/45 (51.1)	26/45 (57.8)	30/45 (66.7)
Rate Difference (%)	-22.2	-15.7	-24.6
p-value	0.047*	0.189	0.029*
Age 60Y+			
Astodrimer [Events/N (%)]	8/29 (27.6)	13/29 (44.8)	13/29 (44.8)
Placebo [Events/N (%)]	18/36 (50.0)	18/36 (50.0)	22/36 (61.1)
Rate Difference (%)	-22.4	-5.2	-16.3
p-value	0.080	0.804	0.219
Age 65Y+			
Astodrimer [Events/N (%)]	4/22 (18.2)	9/22 (40.9)	9/22 (40.9)
Placebo [Events/N (%)]	14/27 (51.9)	13/27 (48.1)	16/27 (59.3)
Rate Difference (%)	-33.7	-7.2	-18.4
p-value	0.019*	0.774	0.256

* $p<0.05$.

Table S11: Proportion of participants who recovered their lost sense of smell and/or taste

	Smell	Taste	Smell or Taste
All ages (mITT)			
Astodrimer [Events/N (%)]	21/33 (63.6)	26/37 (70.3)	27/43 (62.8)
Placebo [Events/N (%)]	31/50 (62.0)	30/50 (60.0)	35/59 (59.3)
Rate Difference (%)	1.6	10.3	3.5
p-value	1.000	0.371	0.838
Age 40Y+			
Astodrimer [Events/N (%)]	14/23 (60.9)	15/24 (62.5)	16/28 (57.1)
Placebo [Events/N (%)]	22/37 (59.5)	23/39 (59.0)	26/45 (57.8)
Rate Difference (%)	1.4	3.5	-0.6
p-value	1.000	1.000	1.000
Age 45Y+			
Astodrimer [Events/N (%)]	12/17 (70.6)	12/20 (60.0)	14/22 (63.6)
Placebo [Events/N (%)]	18/32 (56.2)	19/33 (57.6)	22/39 (56.4)
Rate Difference (%)	14.3	2.4	7.2
p-value	0.373	1.000	0.787
Age 50Y+			
Astodrimer [Events/N (%)]	9/13 (69.2)	9/16 (56.2)	10/17 (58.8)
Placebo [Events/N (%)]	14/26 (53.8)	16/28 (57.1)	18/33 (54.5)
Rate Difference (%)	15.4	-0.9	4.3
p-value	0.495	1.000	1.000
Age 55Y+			
Astodrimer [Events/N (%)]	7/10 (70.0)	8/14 (57.1)	8/14 (57.1)
Placebo [Events/N (%)]	12/23 (52.2)	14/26 (53.8)	16/30 (53.3)
Rate Difference (%)	17.8	3.3	3.8
p-value	0.455	1.000	1.000
Age 60Y+			
Astodrimer [Events/N (%)]	6/7 (85.7)	8/11 (72.7)	8/11 (72.7)
Placebo [Events/N (%)]	10/18 (55.6)	10/18 (55.6)	12/22 (54.5)
Rate Difference (%)	30.2	17.2	18.2
p-value	0.355	0.449	0.456
Age 65Y+			
Astodrimer [Events/N (%)]	2/3 (66.7)	4/7 (57.1)	4/7 (57.1)
Placebo [Events/N (%)]	8/14 (57.1)	7/13 (53.8)	9/16 (56.2)
Rate Difference (%)	9.5	3.3	0.9
p-value	1.000	1.000	1.000

Table S12: Summary of adverse events

	Astodrimer (N=109)	Placebo (N=113)	Total (N=222)
**Number of participants with:**			
Any AE	16 (14.7) 22	15 (13.3) 23	31 (14.0) 45
Any severe AE	1 (0.9) 1	1 (0.9) 2	2 (0.9) 3
Any non-related AE	14 (12.8) 19	14 (12.4) 22	28 (12.6) 41
Any related AE (ADE)	3 (2.8) 3	1 (0.9) 1	4 (1.8) 4
Any AE leading to discontinuation from study product administration	1 (0.9) 1	2 (1.8) 3	3 (1.4) 4
Any AE leading to discontinuation from the investigation	1 (0.9) 1	2 (1.8) 3	3 (1.4) 4
Any serious AE	1 (0.9) 1	1 (0.9) 2	2 (0.9) 3
Any serious ADE	0 (0.0) 0	0 (0.0) 0	0 (0.0) 0
Any AE resulting in death	0 (0.0) 0	0 (0.0) 0	0 (0.0) 0
**AEs by System Organ Class / Preferred Term**			
Respiratory, thoracic and mediastinal disorders	6 (5.5) 6	3 (2.7) 3	9 (4.1) 9
Epistaxis	3 (2.8) 3	1 (0.9) 1	4 (1.8) 4
Nasal discomfort	2 (1.8) 2	1 (0.9) 1	3 (1.4) 3
Dysphonia	0 (0.0) 0	1 (0.9) 1	1 (0.5) 1
Nasal congestion	1 (0.9) 1	0 (0.0) 0	1 (0.5) 1
Gastrointestinal disorders	2 (1.8) 2	5 (4.4) 6	7 (3.2) 8
Abdominal pain upper	0 (0.0) 0	2 (1.8) 2	2 (0.9) 2
Abdominal pain	1 (0.9) 1	0 (0.0) 0	1 (0.5) 1
Constipation	0 (0.0) 0	1 (0.9) 1	1 (0.5) 1
Gingival pain	0 (0.0) 0	1 (0.9) 1	1 (0.5) 1
Oral disorder	0 (0.0) 0	1 (0.9) 1	1 (0.5) 1
Oral pain	1 (0.9) 1	0 (0.0) 0	1 (0.5) 1
Toothache	0 (0.0) 0	1 (0.9) 1	1 (0.5) 1
Ear and labyrinth disorders	4 (3.7) 4	2 (1.8) 2	6 (2.7) 6
Ear discomfort	2 (1.8) 2	0 (0.0) 0	2 (0.9) 2
Ear pain	1 (0.9) 1	1 (0.9) 1	2 (0.9) 2
Tinnitus	1 (0.9) 1	1 (0.9) 1	2 (0.9) 2
Nervous system disorders	2 (1.8) 3	2 (1.8) 2	4 (1.8) 5
Somnolence	2 (1.8) 2	0 (0.0) 0	2 (0.9) 2
Dysgeusia	0 (0.0) 0	1 (0.9) 1	1 (0.5) 1
Psychomotor hyperactivity	1 (0.9) 1	0 (0.0) 0	1 (0.5) 1
Syncope	0 (0.0) 0	1 (0.9) 1	1 (0.5) 1
General disorders and administration site conditions	0 (0.0) 0	3 (2.7) 3	3 (1.4) 3
Application site reaction	0 (0.0) 0	1 (0.9) 1	1 (0.5) 1
Chest discomfort	0 (0.0) 0	1 (0.9) 1	1 (0.5) 1
Facial discomfort	0 (0.0) 0	1 (0.9) 1	1 (0.5) 1
Infections and infestations	1 (0.9) 1	2 (1.8) 2	3 (1.4) 3
Gingivitis	0 (0.0) 0	1 (0.9) 1	1 (0.5) 1
Pneumonia	1 (0.9) 1	0 (0.0) 0	1 (0.5) 1
Urosepsis	0 (0.0) 0	1 (0.9) 1	1 (0.5) 1
Musculoskeletal and connective tissue disorders	2 (1.8) 2	1 (0.9) 1	3 (1.4) 3
Arthralgia	0 (0.0) 0	1 (0.9) 1	1 (0.5) 1
Back pain	1 (0.9) 1	0 (0.0) 0	1 (0.5) 1
Pain in extremity	1 (0.9) 1	0 (0.0) 0	1 (0.5) 1
Eye disorders	1 (0.9) 1	1 (0.9) 1	2 (0.9) 2
Eye pain	1 (0.9) 1	0 (0.0) 0	1 (0.5) 1

	Astodrimer (N=109)	Placebo (N=113)	Total (N=222)
Eye swelling	0 (0.0) 0	1 (0.9) 1	1 (0.5) 1
Renal and urinary disorders	1 (0.9) 1	1 (0.9) 1	2 (0.9) 2
Chromaturia	0 (0.0) 0	1 (0.9) 1	1 (0.5) 1
Renal pain	1 (0.9) 1	0 (0.0) 0	1 (0.5) 1
Metabolism and nutrition disorders	0 (0.0) 0	1 (0.9) 1	1 (0.5) 1
Diabetic ketoacidosis	0 (0.0) 0	1 (0.9) 1	1 (0.5) 1
Psychiatric disorders	1 (0.9) 1	0 (0.0) 0	1 (0.5) 1
Sleep disorder	1 (0.9) 1	0 (0.0) 0	1 (0.5) 1
Skin and subcutaneous tissue disorders	0 (0.0) 0	1 (0.9) 1	1 (0.5) 1
Yellow skin	0 (0.0) 0	1 (0.9) 1	1 (0.5) 1
Vascular disorders	1 (0.9) 1	0 (0.0) 0	1 (0.5) 1
Hot flush	1 (0.9) 1	0 (0.0) 0	1 (0.5) 1

Data is n (%) m, where n=Number of participants experiencing the AE; %=Percentage of participants experiencing the AE; and m=Number of AEs.

Verbatim terms coded using MedDRA (version 26.1)

Supplementary figures

Time to negative RT-qPCR test

Figure S1: Time in days from baseline to negative RT-qPCR (All ages)

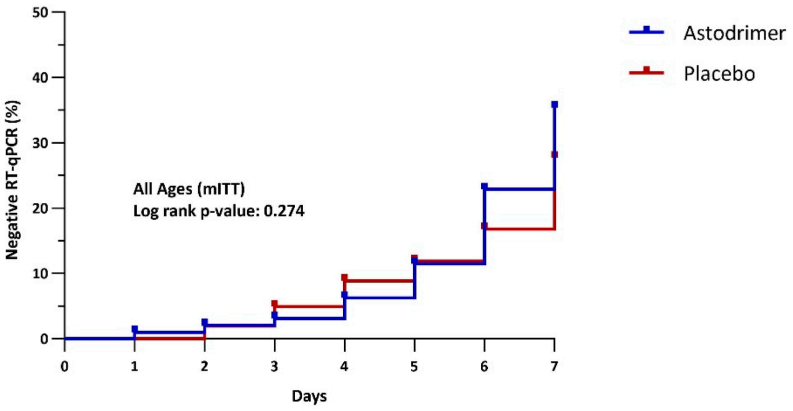

Figure S2: Time in days from baseline to negative RT-qPCR (40Y+)

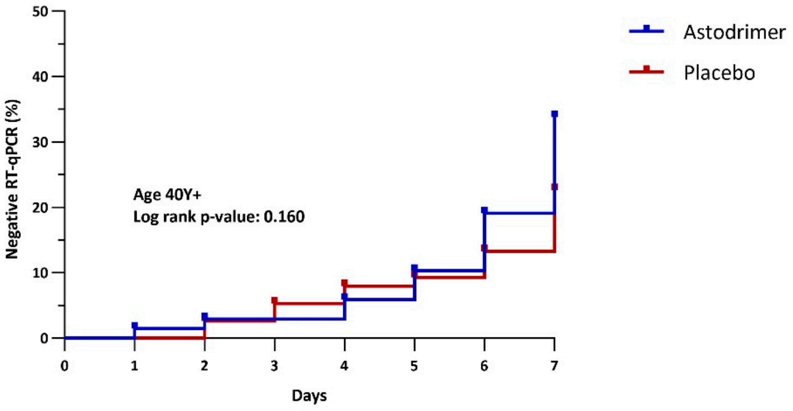

Figure S3: Time in days from baseline to negative RT-qPCR (45Y+)

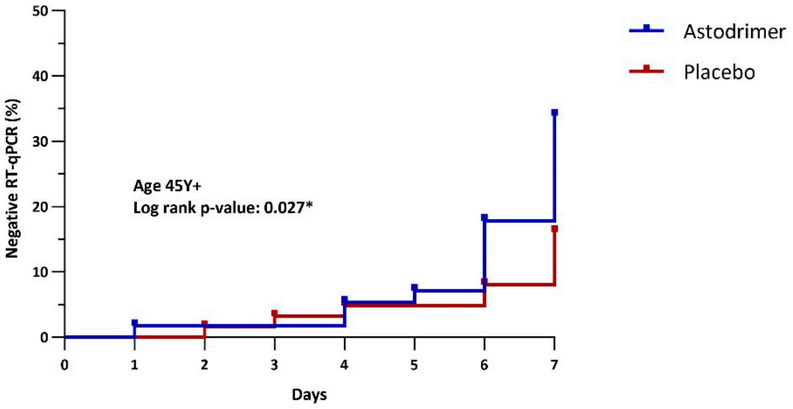

Figure S4: Time in days from baseline to negative RT-qPCR (50Y+)

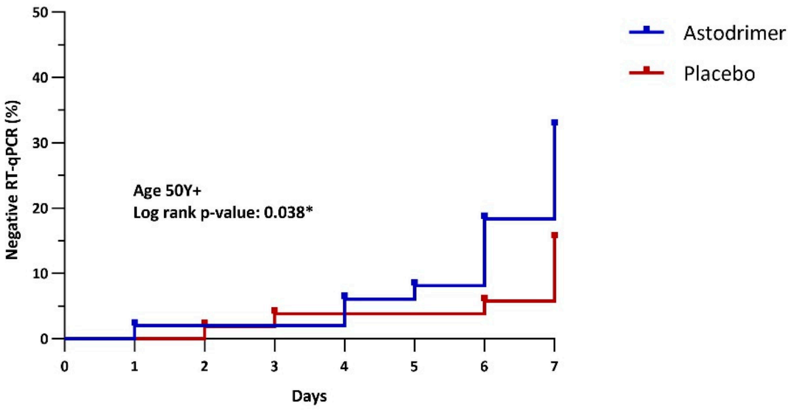

Figure S5: Time in days from baseline to negative RT-qPCR (55Y+)

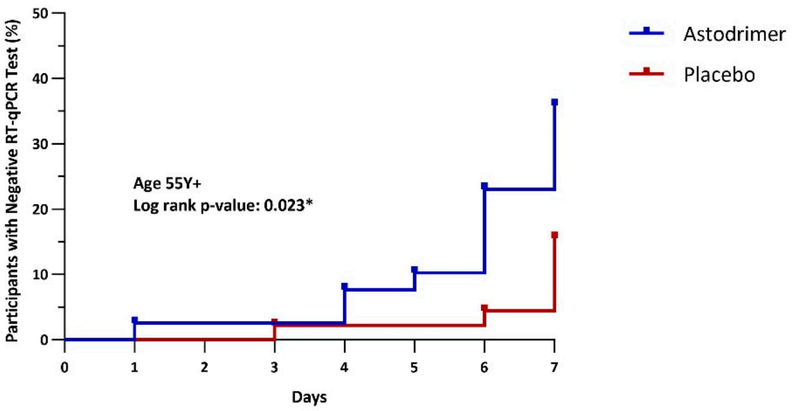

Figure S6: Time in days from baseline to negative RT-qPCR (60Y+)

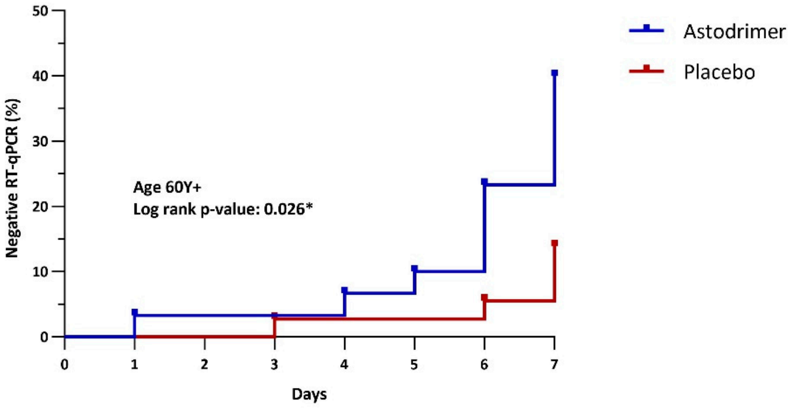

Figure S7: Time in days from baseline to negative RT-qPCR (65Y+)

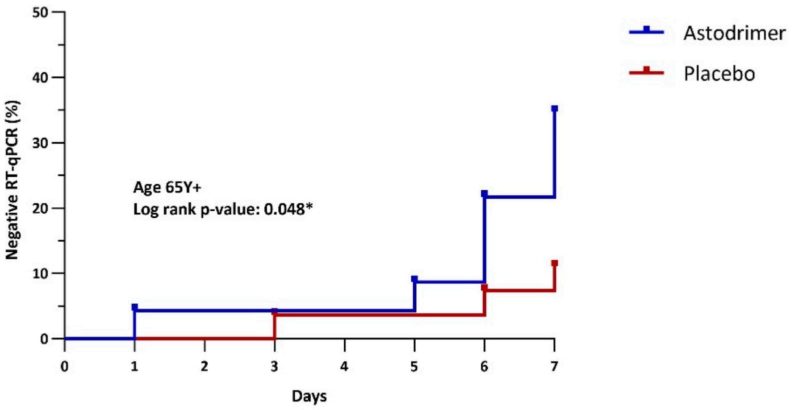

Peak post-baseline nasal swab SARS-CoV-2 RNA load

Figure S8: Peak post-baseline nasal swab SARS-CoV-2 RNA load

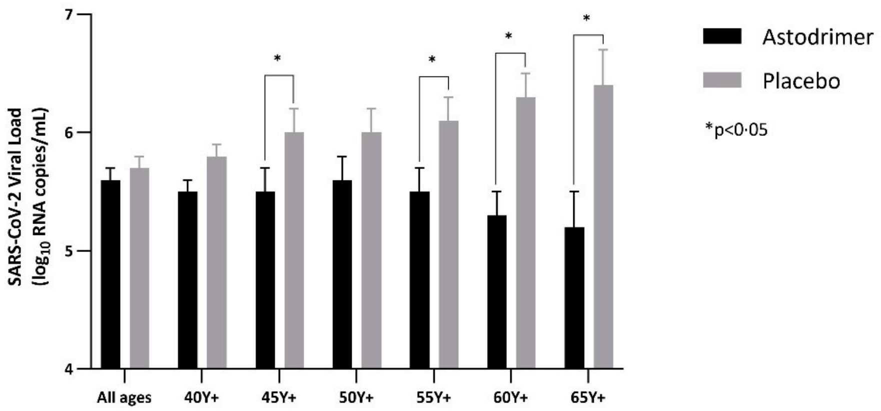

SARS-CoV-2 RNA load at each determination

Figure S9: SARS-CoV-2 RNA load at each determination (All ages)

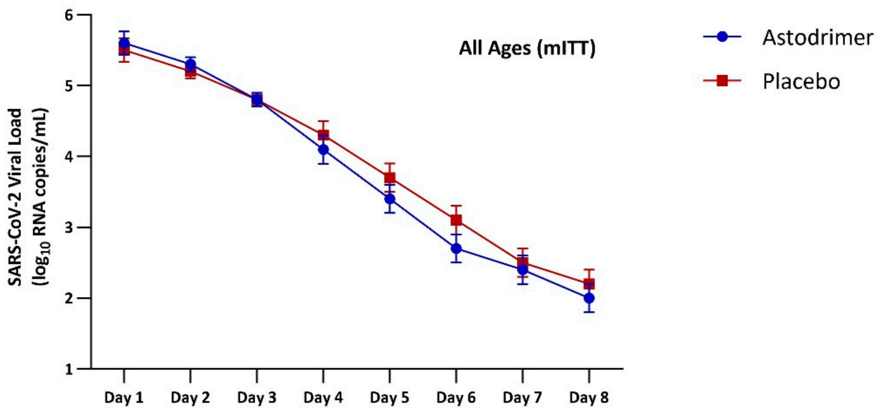

Figure S10: SARS-CoV-2 RNA load at each determination (40Y+)

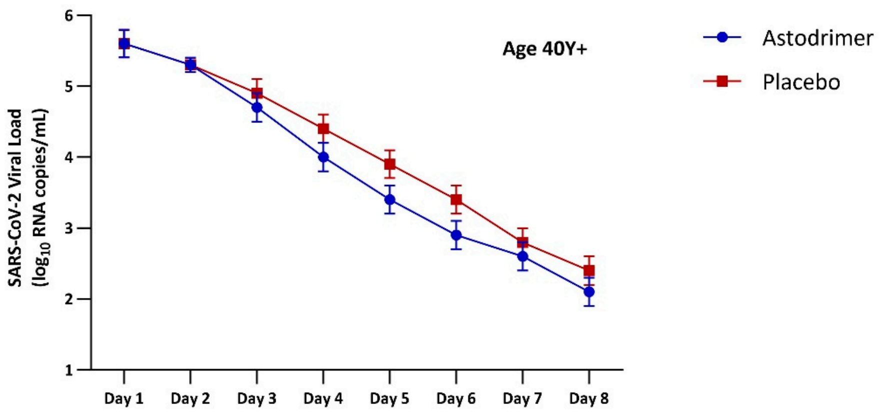

Figure S11: SARS-CoV-2 RNA load at each determination (45Y+)

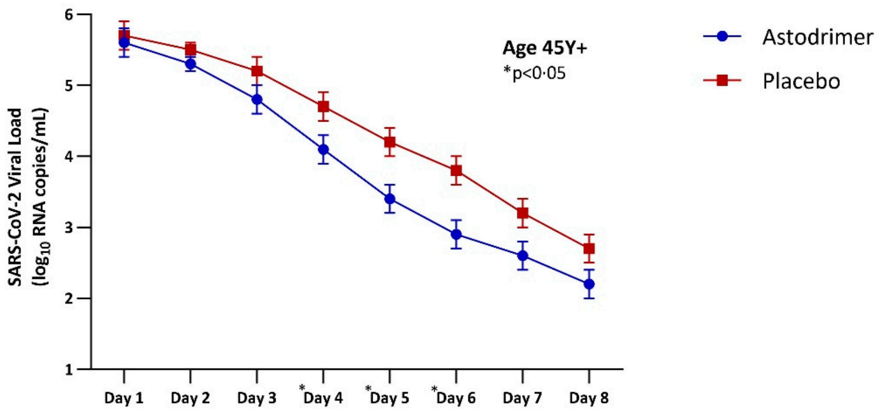

Figure S12: SARS-CoV-2 RNA load at each determination (50Y+)

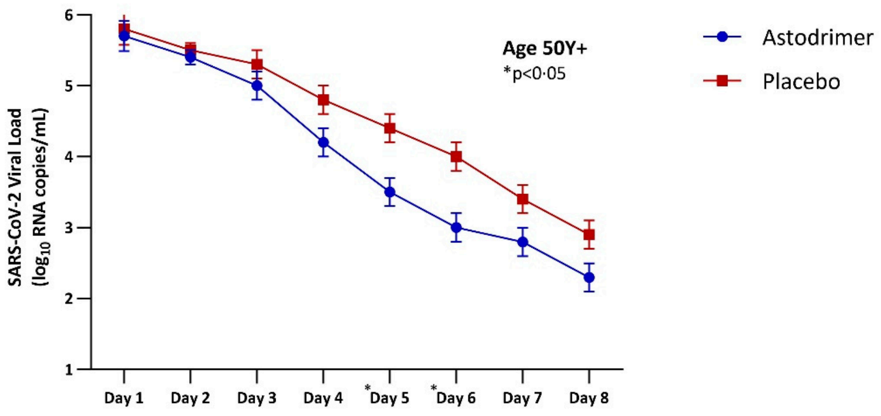

Figure S13: SARS-CoV-2 RNA load at each determination (55Y+)

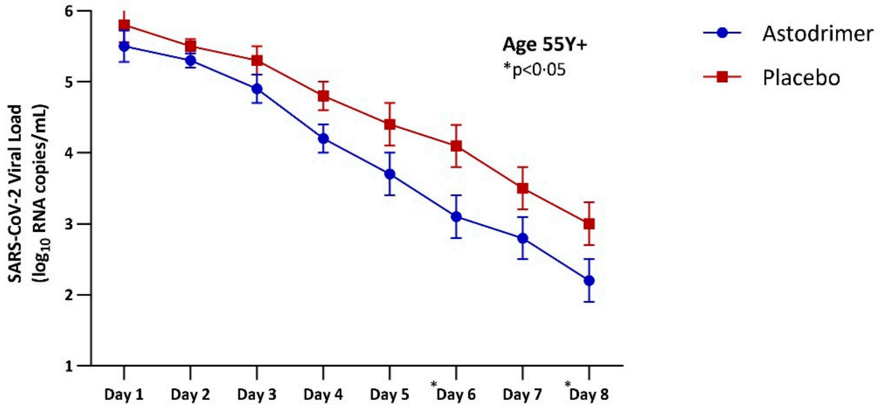

Figure S14: SARS-CoV-2 RNA load at each determination (60Y+)

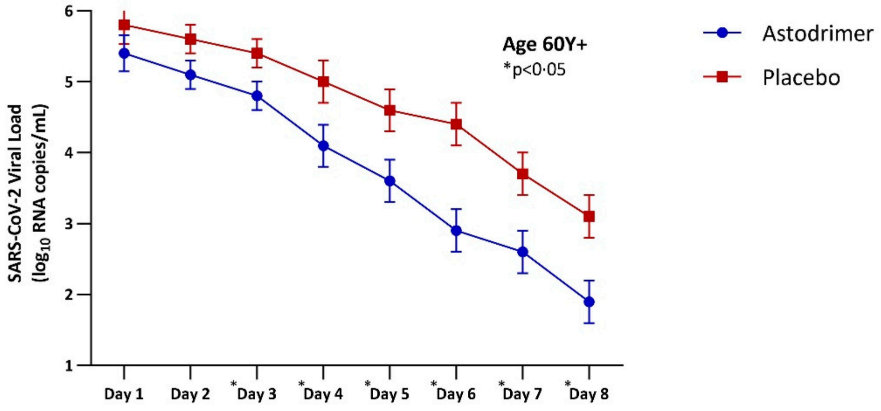

Figure S15: SARS-CoV-2 RNA load at each determination (65Y+)

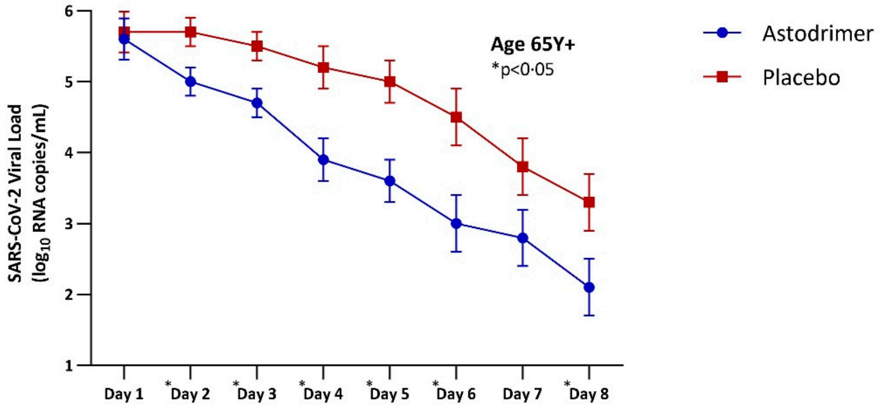

Mean change in SARS-CoV-2 RNA load at each determination relative to the baseline determination

Figure S16: Mean SARS-CoV-2 RNA load change at each determination relative to baseline (All ages)

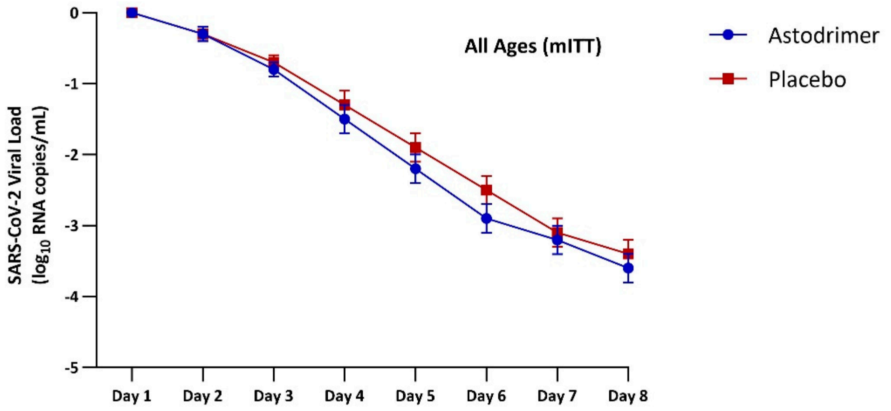

Figure S17: Mean SARS-CoV-2 RNA load change at each determination relative to baseline (40Y+)

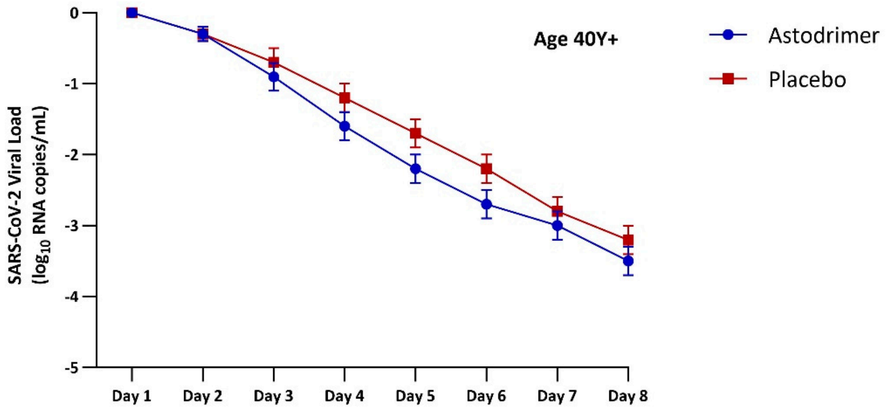

Figure S18: Mean SARS-CoV-2 RNA load change at each determination relative to baseline (45Y+)

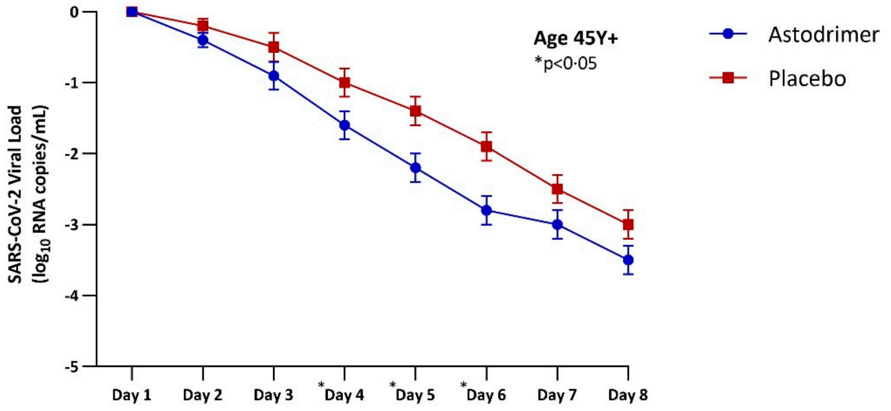

Figure S19: Mean SARS-CoV-2 RNA load change at each determination relative to baseline (50Y+)

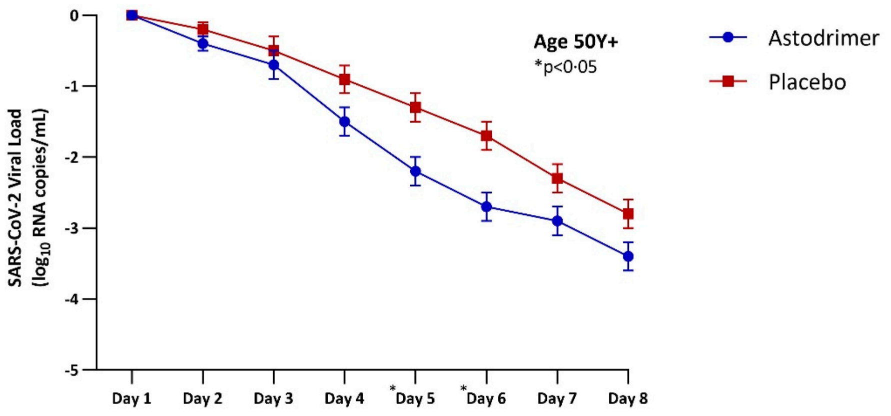

Figure S20: Mean SARS-CoV-2 RNA load change at each determination relative to baseline (55Y+)

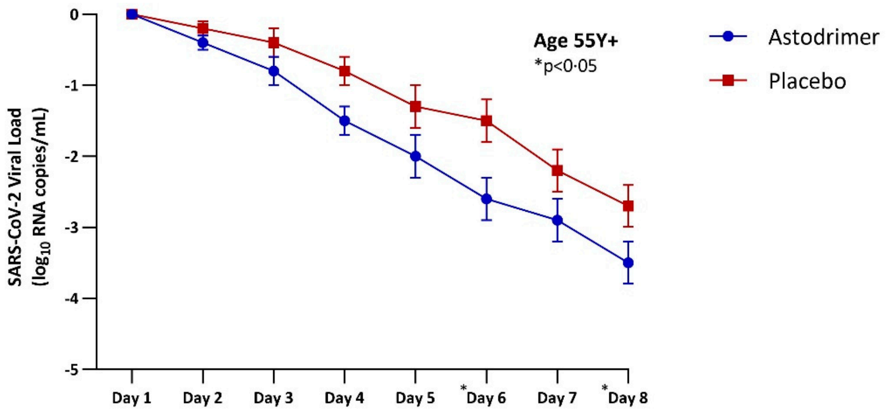

Figure S21: Mean SARS-CoV-2 RNA load change at each determination relative to baseline (60Y+)

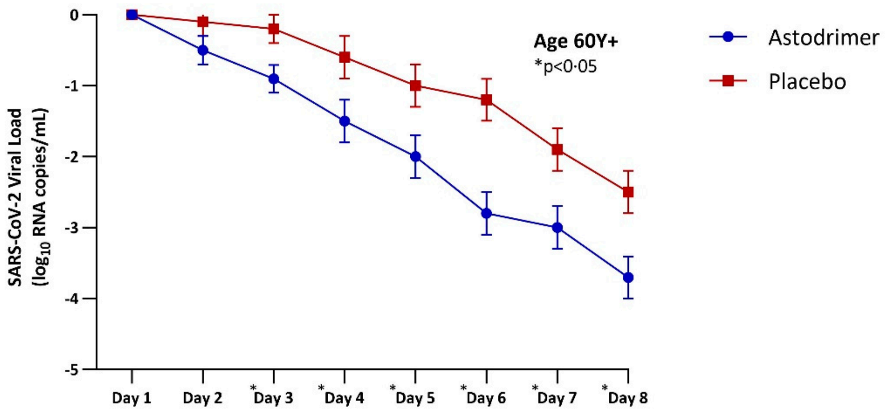

Figure S22: Mean SARS-CoV-2 RNA load change at each determination relative to baseline (65Y+)

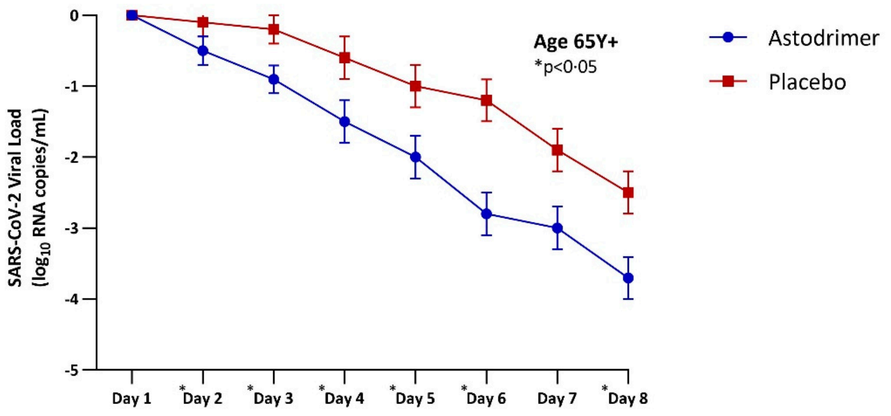

Supplement: Supplementary file 1 [file pharmaceutics-16-01173-s001.zip › pharmaceutics-3185477-supplementary.pdf]
